# N-(3-oxo-acyl) homoserine lactone inhibits tumor growth independent of Bcl-2 proteins

**DOI:** 10.18632/oncotarget.6827

**Published:** 2016-01-07

**Authors:** Guoping Zhao, Aaron M. Neely, Christian Schwarzer, Huayi Lu, Aaron G. Whitt, Nicole S. Stivers, Joseph A. Burlison, Carl White, Terry E. Machen, Chi Li

**Affiliations:** ^1^ Molecular Targets Program, University of Louisville, Louisville, KY 40202, USA; ^2^ Structural Biology Program, James Graham Brown Cancer Center, Departments of Medicine, Pharmacology and Toxicology, University of Louisville, Louisville, KY 40202, USA; ^3^ Department of Molecular and Cell Biology, University of California, Berkeley, CA 94720, USA; ^4^ Second Hospital of Jilin University, Changchun, Jilin Province, P.R. China 130041; ^5^ Department of Physiology and Biophysics, Rosalind Franklin University of Medicine and Science, North Chicago, IL 60064, USA; ^6^ Institute of Technical Biology and Agriculture Engineering, Hefei Institutes of Physical Science, Chinese Academy of Sciences, Hefei, Anhui Province, P.R. China 230031

**Keywords:** homoserine lactone, Bcl-2, Bak, Bax, tumor

## Abstract

*Pseudomonas aeruginosa* produces N-(3-oxododecanoyl)-homoserine lactone (C12) as a quorum-sensing molecule for bacterial communication. C12 has also been reported to induce apoptosis in various types of tumor cells. However, the detailed molecular mechanism of C12-triggerred tumor cell apoptosis is still unclear. In addition, it is completely unknown whether C12 possesses any potential therapeutic effects *in vivo*. Our data indicate that, unlike most apoptotic inducers, C12 evokes a novel form of apoptosis in tumor cells through inducing mitochondrial membrane permeabilization independent of both pro- and anti-apoptotic Bcl-2 proteins. Importantly, C12 inhibits tumor growth in animals regardless of either pro- or anti-apoptotic Bcl-2 proteins. Furthermore, opposite to conventional chemotherapeutics, C12 requires paraoxonase 2 (PON2) to exert its cytotoxicity on tumor cells *in vitro* and its inhibitory effects on tumor growth *in vivo*. Overall, our results demonstrate that C12 inhibits tumor growth independent of both pro- and anti-apoptotic Bcl-2 proteins, and through inducing unique apoptotic signaling mediated by PON2 in tumor cells.

## INTRODUCTION

Quorum sensing is a bacterial intercellular communication system that constitutively produces, releases, and detects small diffusible autoinducers that are similar to mammalian hormones [1]. The gram-negative human pathogen *Pseudomonas aeruginosa* uses N-(3-oxododecanoyl)-L-homoserine lactone (C12) as a quorum sensing signal [2, 3].

Recent studies have shown that C12 is not only important in regulating bacterial virulence genes but also interacts with eukaryotic cells and modulates cell physiology, such as triggering cell death [4]. C12 has been demonstrated to cause apoptosis in a variety of cancer cells [5–8]. C12 induces apoptosis through inhibiting the phosphatidylinositide 3-kinases and Akt/PKB pathway and diminishing STAT3 activities in breast carcinoma cells [5]. In pancreatic carcinoma cells, C12 also triggers apoptotic signaling and inhibits cell migration [6]. C12 decreases the expression of thymidylate synthase and enhances the activity of chemotherapeutic agents, 5-fluorouracil (5-FU), Tomudex and Taxol in colorectal and prostate cancer cells. Recently, a derivative of C12, 3-oxo-12-phenyldodecanoyl-L-homoserine lactone, has been identified as another cancer cell growth inhibitor [8]. Comparative SAR analysis demonstrates that long acyl side chains with a 3-oxo substitution are essential for C12′s anti-cancer effect [8]. In light of its function of triggering tumor cell death, C12 displays promise as a cancer treatment. However, detailed apoptotic signaling of C12 remain unclear and whether C12 cytotoxicity *in vitro* is relevant to tumor growth *in vivo* has never been studied.

Resistance toward apoptosis is a hallmark of most, perhaps all, types of human cancer [9, 10]. Bcl-2 proteins are the major regulators of apoptotic signaling pathways and can be classified into anti-apoptotic and pro-apoptotic groups. Anti-apoptotic Bcl-2 proteins such as Bcl-2 are considered to protect against mitochondrial outer membrane permeabilization (MOMP) during apoptosis, whereas pro-apoptotic Bcl-2 members such as Bax and Bak promote MOMP [11, 12]. The expression of individual Bcl-2 proteins in different types of cancer has been used as an independent prognostic marker [10]. Studies in various human tumors showed that loss of Bax expression, or increased expression of Bcl-2, are associated with their resistance to chemotherapy [13–15]. Accordingly, one strategy for cancer therapy is to identify agonists that activate apoptotic pathway independent of Bcl-2 proteins in tumor cells [16–18].

As a lactone, C12 is known to be hydrolyzed into a carboxylic acid by the lactonase paraoxonase 2 (PON2), which belongs to a gene family (PON1, PON2 and PON3) with Ca^2+^-dependent lactonase and arylesterase activities [19, 20]. In murine airway epithelia, PON2 attenuates *Pseudomonas aeruginosa* quorum sensing by inactivating C12 [21]. PON2 and PON3 also display anti-oxidant and anti-inflammatory functions [22–24]. The detailed mechanism by which PON2 exerts these effects remains unknown. Importantly, PON2 expression is markedly elevated in several human non-small cell lung carcinoma (NSCLC) cell lines, which is associated with resistance to classical anticancer drugs like doxorubicin or cisplatin [23, 24]. In contrast, overexpression of PON2 promotes C12-induced apoptosis in MEFs and HEK293T cells [25].

To gain insights into the mechanism of C12-evoked tumor cell apoptosis, we evaluated the cytotoxic effects of C12 on tumor cells *in vitro* and the inhibitory effects of C12 on tumor growth *in vivo*. The role of pro- and anti-apoptotic Bcl-2 and PON2 proteins in C12-induced apoptosis was systematically examined. C12 inhibited the tumor growth through inducing apoptosis in a PON2-dependent but not Bcl-2 protein-dependent manner. Our study reveals a unique anti-tumor function of C12, which may lead to the development of new therapeutic agents for cancer.

## RESULTS

### C12 inhibits lung tumor growth and induces tumor cell apoptosis *in vivo*

**Figure 1:**
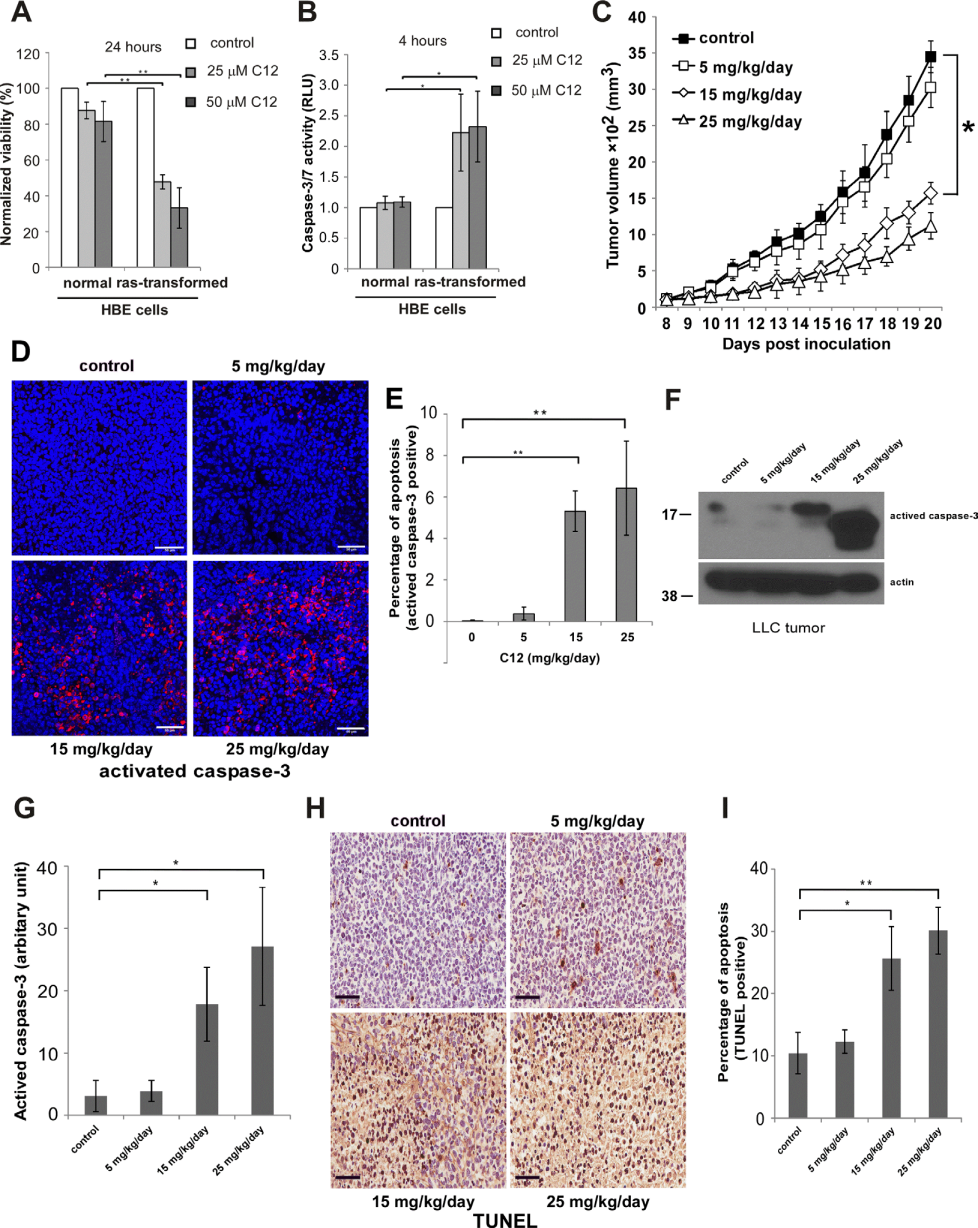
C12 inhibits LLC tumor growth and induces tumor cell apoptosis *in vivo* in a dose-dependent fashion (**A**–**B**) Cytotoxicity of C12 is affected by oncogenic transformation. C12's effects on HBE cell viability (A) and caspase-3/7 activation (B) were examined. All data shown are mean ± standard deviation of 3 independent experiments. Asterisk indicates *P* < 0.05 (*) or *P* < 0.01 (**) by student's unpaired *t* test. (**C**) The inhibitory effects of C12 on the growth of LLC tumors were studied. Tumors were measured daily and tumor tissues were removed at the end of treatments. Data are shown as mean ± standard deviation of tumor volumes of 7 animals in either vehicle control or C12-treated group. Asterisk indicates *P* < 0.05 (*) by student's unpaired *t* test. (**D**) Apoptotic cells in tumor sections were detected by immunofluorescence staining of activated caspase-3. Representative images of tumor sections are shown. Scale bar, 50 μm. (**E**) The percentage of activated caspase-3 shown in (D) was quantified using ImageJ software (NIH). Data are mean ± standard deviation of three independent tumor sections. Asterisk indicates *P* < 0.01 (**) by student's unpaired *t* test. (**F**) Expression of activated caspase-3 in tumor tissues was analyzed by western blot. (**G**) The relative expression levels of activated caspase-3 shown in (F) were quantified by measuring intensities of western blot signals using ImageJ software and presented as arbitrary units. Data are mean ± standard deviation of three independent tumor samples. Asterisk indicates *P* < 0.05 (*) by student's unpaired *t* test. (**H**) TUNEL staining of apoptotic cells in control or C12-treated tumor sections. Representative images are shown. Scale bar, 60 μm. (**I**) The percentage of apoptotic cells shown in (H) was quantified using ImageJ software. Data are mean ± standard deviation of three independent tumor sections. Asterisk indicates *P* < 0.05 (*) or *P* < 0.01 (**) by student's unpaired *t* test.

The cytotoxic effects of C12 on tumor cells have been reported previously [5–8, 26], but whether they are selective for transformed cells was unknown. To investigate whether oncogenic transformation influences the cytotoxicity of C12, we studied normal human bronchia/tracheal epithelial (NHBE) and corresponding HBE immortalized and transformed successively by telomerase, SV40 large T antigen and activated Ras (H-ras V12) [27–29]. This is a well-established epithelial cell malignant transformation system related to human lung cancer. Following C12 treatment, transformed HBE exhibited higher levels of cell death (Figure 1A and Supplementary Figure 1A) and caspase-3/7 activation (Figure 1B and Supplementary Figure 1B) compared with their untransformed counterparts, indicating that C12 induces apoptosis preferentially in transformed cells.

To investigate the relevance of C12 cytotoxicity on transformed cells to tumor growth in animals, we examined the effects of C12 on the growth of established Lewis Lung Carcinoma (LLC) tumors. As shown in Figure 1C, transplanted tumors grew much more slowly in C12-treated mice than in vehicle-treated mice, revealing a dose-dependent anti-tumor activity of C12 as a single agent. No significant changes of body weight and organ weight (spleen, kidney, liver, heart and lung) were observed for C12-treated C57BL/6 mice (Supplementary Figure 2A and 2C), showing no evidence of significant toxicities of C12 administration. To test the involvement of apoptosis in tumor growth inhibition, established LLC tumors from mice were analyzed for caspase-3 activation through immunofluorescence staining (Figure 1D–1E), western blot (Figure 1F–1G) and TUNEL staining (Figure 1H–1I). The percentage of TUNEL-positive cells and the levels of activated caspase-3 were higher in tumors from C12–treated mice than in those of the control group, suggesting that apoptosis is involved in the inhibitory activity of C12 *in vivo*.

In order to explore the therapeutic potential of C12, we studied the combinatorial effects of C12 and therapeutic drugs. Cell death of human lung tumor cells A549 and NCI-H1299 treated with various concentrations of C12 and the inhibitor of Bcl-2 and Bcl-x_L_ ABT-737 at a constant dose ratio were measured, and the combination index (CI) values were determined (Supplementary Figure 3). As shown in Supplementary Figure 3C and 3F, in the range of tested drug concentrations, CI values were smaller than 1, indicating that C12 and ABT-737 exhibited synergistic cytotoxic effects on A549 cells and NCI-H1299 cells.

### Anti-apoptotic Bcl-2 proteins does not affect C12 cytotoxicity

**Figure 2:**
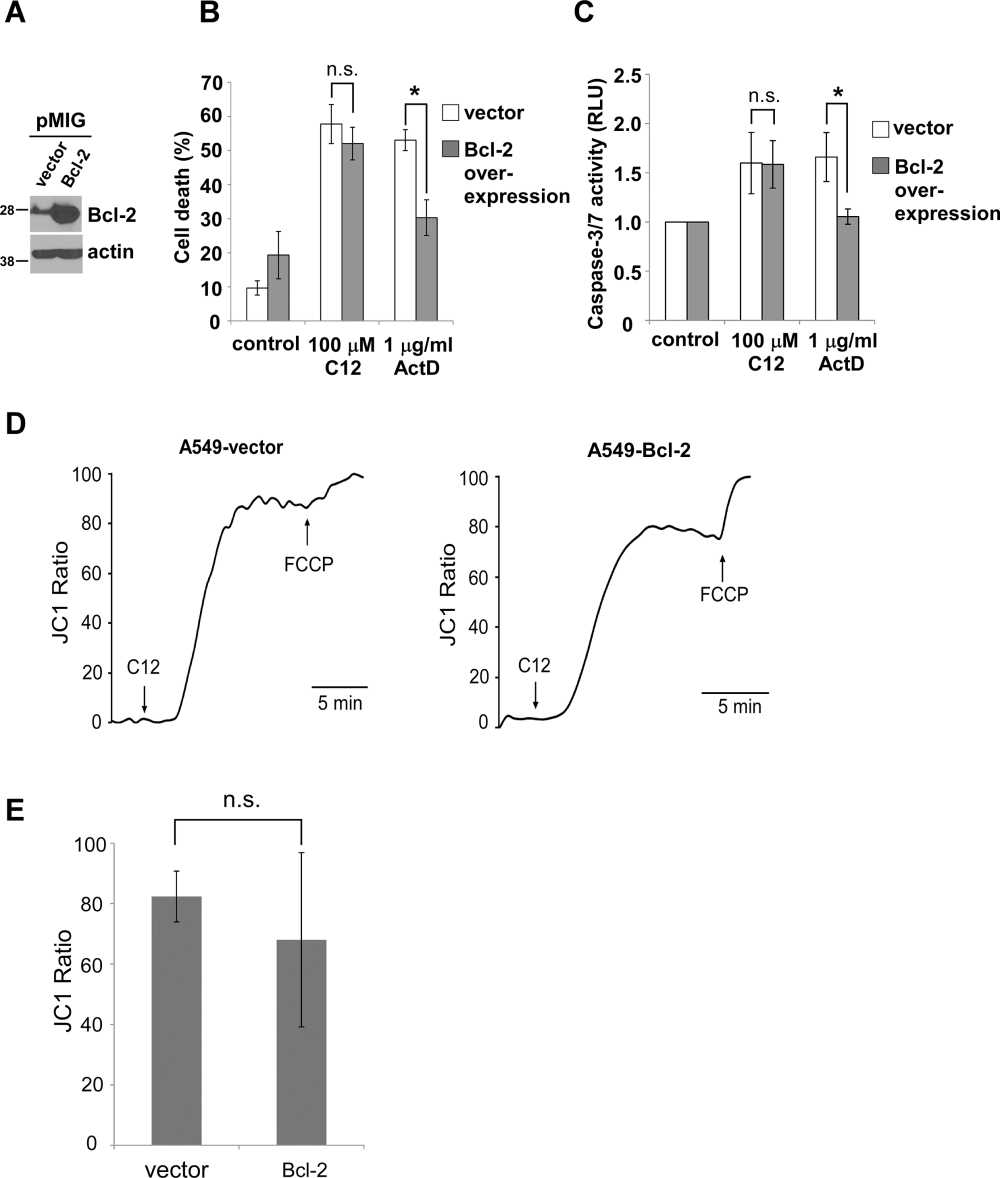
C12 induces tumor apoptotic cell death independent of anti-apoptotic Bcl-2 proteins (**A**) Retrovirally overexpressed Bcl-2 in A549 cells was examined by western blot. (**B**) Cell viability was measured 48 hours after C12 or actinomycin D (ActD) exposure. (**C**) Caspase-3/7 activities were determined following 2 hour exposure to C12 and 24 hour exposure to actinomycin D. (**D**) C12's effect on mitochondrial membrane potential is independent of Bcl-2. A549-vector and A549-Bcl-2-overexpressing cells were loaded with JC1, and its fluorescence was measured using imaging microscopy during the treatment with 100 μM C12 and 5 μM FCCP. Typical results from three independent experiments are shown. (**E**) C12 caused equivalent depolarization of mitochondrial potential in vector and Bcl-2-overexpressing A549 cells. All data are shown as mean ± standard deviation of three independent experiments. Asterisks indicate *P* < 0.05 (*); ns, no significant by student's unpaired *t* test.

Anti-apoptotic Bcl-2 proteins are frequently overexpressed in human cancers and associated with chemotherapeutic resistance and relapse [30]. To investigate the involvement of anti-apoptotic Bcl-2 proteins in C12-induced human tumor cell apoptosis, Bcl-2, Mcl-1 and Bcl-x_L_ were stably overexpressed in A549 cells respectively by retroviral infection (Figure 2A and Supplementary Figure 4A and 4D). The anti-tumor drug actinomycin D caused less cell death and caspase-3/7 activation in Bcl-2-, Mcl-1- or Bcl-x_L_-overexpressing cells than in cells expressing the empty vector (Figure 2B–2C and Supplementary Figure 4B–4C and 4E–4F). In contrast, C12 induced similar levels of cell death and caspase-3/7 activation in cells overexpressing Bcl-2, Mcl-1 or Bcl-x_L_ and the vector control cells (Figure 2B–2C and Supplementary Figure 4B–4C and 4E–4F). To further explore the involvement of Mcl-1 in mediating C12-induced cytotoxicity, we examined A549 cells in which Mcl-1 expression was stably reduced by shRNA [31]. Actinomycin D but not C12 caused more cell death and caspase-3/7 activation in Mcl-1-knockdown cells than the cells expressing the control scramble shRNA (Supplementary Figure 4G–4H), further validating that Mcl-1 expression is not vital for C12-induced apoptotic signaling.

MOMP has been recognized to be a “no-return” step during mitochondria-based apoptotic signaling [32]. To further investigate C12-initiated apoptosis in tumor cells, we studied the involvement of Bcl-2 in two key events of MOMP: depolarization of mitochondrial membrane potential (Δψ_mito_) and cytochrome c release. Depolarization of Δψ_mito_ was evaluated by determining the changes in fluorescence of the voltage-dependent dye JC1 released from mitochondria into the cytosol and nucleus. Within minutes of C12 exposure, mitochondria in A549-vector cells and A549-Bcl-2 overexpressing cells were depolarized to the same degree (Figure 2D–2E). While depolarizing Δψ_mito_ quickly, C12 did not appear to affect mitochondrial morphology with mitochondrial interconnectivity determined by mean area/perimeter ratio and mitochondrial elongation measured by inverse circularity, indicating that mitochondrial fission/fusion is unlikely involved in the initiation of MOMP in cells exposed to C12 (Supplementary Videos 1–4 and Supplementary Figure 5). Moreover, C12 caused cytochrome c release from mitochondria in both A549-vector and Bcl-2 or Mcl-1 over-expressing A549 cells (Supplementary Figure 6A–6B). Taken together, these results indicate that overexpression of anti-apoptotic Bcl-2 proteins reduces apoptosis in response to the classical anti-tumor drug actinomycin D but fails to provide protection against C12.

### C12 blocks tumor growth independent of Bcl-2 protein

**Figure 3:**
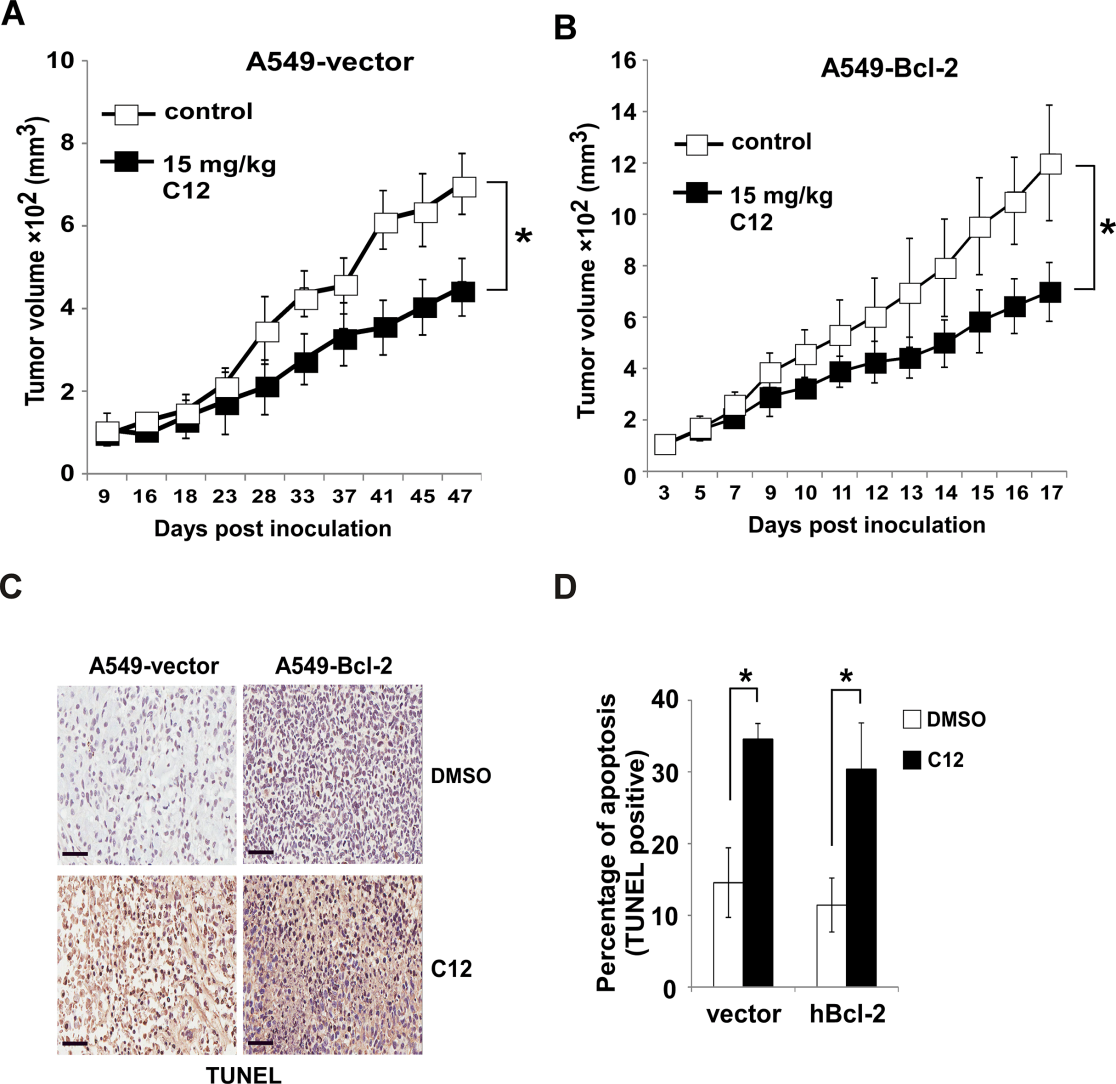
C12 inhibits tumor growth independent of Bcl-2 (**A**–**B**) Growth of A549-vector (A) and A549-Bcl-2-overexpressing tumors (B) in athymic nude mice treated with the vehicle control or C12 (15 mg/kg/day). Data are shown as mean ± standard deviation of tumor volumes of 8 animals in either vehicle control (DMSO) or C12-treated groups. (**C**) Apoptotic cells in tumor sections were identified by TUNEL assay. Representative images of tumor sections from DMSO- and C12-treated mice are shown. Scale bar, 60 μm. (**D**) Summary of the data shown in (C). More apoptotic cells were detected in tumors of C12-treated mice than those of vehicle control mice. Mean ± standard deviation of three independent tumor sections. For all the data, asterisk indicates *P* < 0.05 (*) by student's unpaired *t* test.

To determine whether anti-apoptotic Bcl-2 protein-independent C12 cytotoxicity is relevant *in vivo*, we examined its effects on the growth of tumors originated from A549 cells with different levels of Bcl-2 expression in athymic nude mice. Similarly to C57BL/6 mice, C12 caused no significant change of body weight and organ weight (spleen, kidney, liver, heart and lung) in C12-treated athymic nude mice (Supplementary Figure 2B and 2D). As shown in Figure 3A–3B, A549–Bcl-2 overexpressing tumors grew much faster than their vector control counterparts, which is consistent with the oncogenic function of Bcl-2 [33]. C12 inhibited the growth of vector control A549 tumors and Bcl-2-overexpressing A549 tumors to a comparable degree. Inhibitory effects of C12 on tumor growth are likely attributed to apoptosis, as more TUNEL-positive cells were detected in C12-treated tumors than vehicle-treated ones regardless of Bcl-2 expression levels (Figure 3C–3D). Overall, these results show that Bcl-2 is not involved in the cytotoxic effects of C12 *in vitro* as well as *in vivo*.

### Bak and Bax are not involved in C12-induced apoptosis

**Figure 4:**
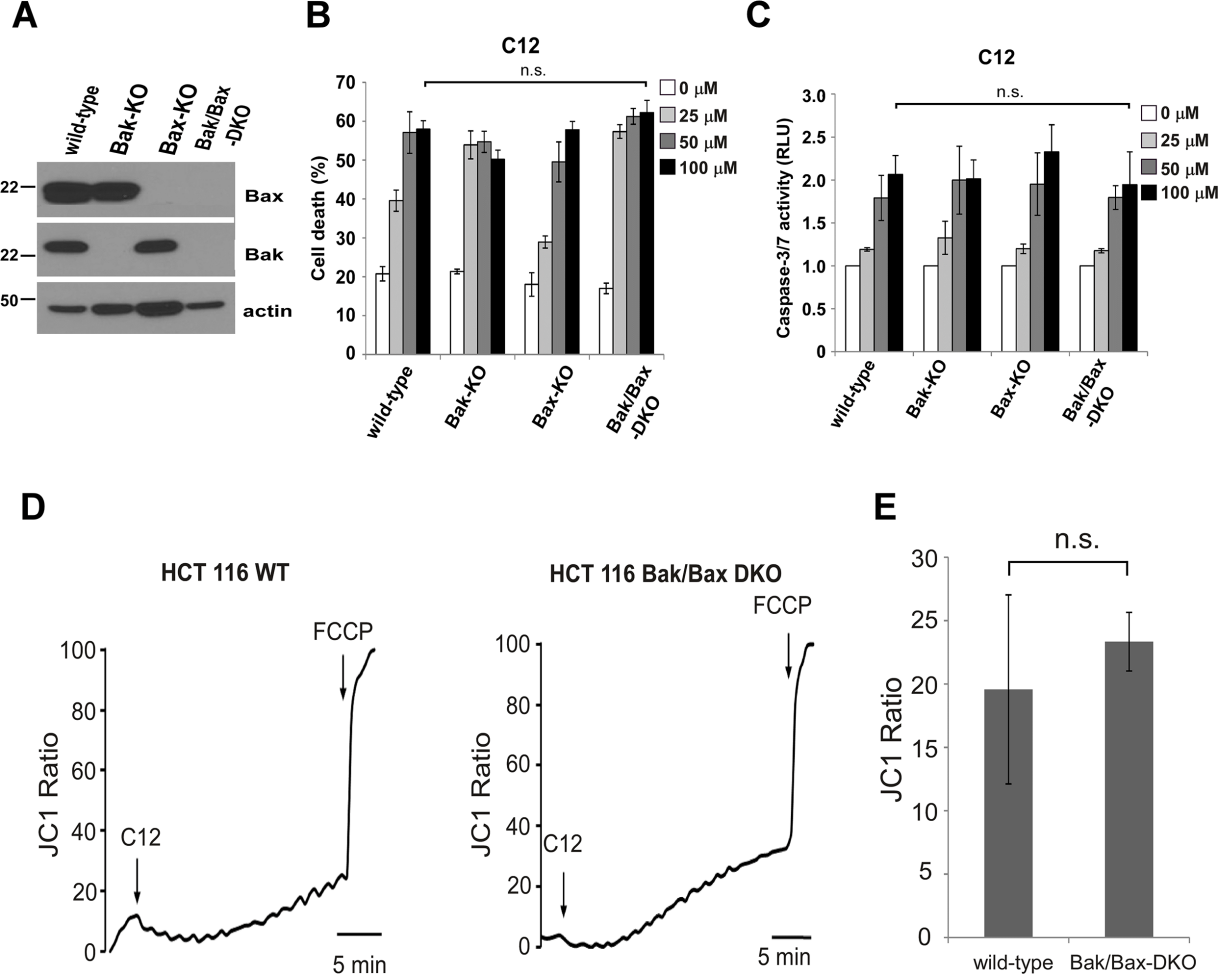
C12-induced tumor cell apoptosis is independent of Bak and Bax (**A**) Bak and Bax expression in the indicated HCT116 cells was examined by western blot. (**B**–**C**) C12 induced similar levels of cell death (B) and caspase-3/7 activation (C) among WT, Bak-KO, Bax-KO and Bak/Bax-DKO HCT116 cells after 24 hours treatment. (**D**) The mitochondrial potential of WT and Bak/Bax-DKO HCT116 cells loaded with JC1 was determined by fluorescent microscopy upon the treatment with 50 μM C12 and 5 μM FCCP. Representative results are shown. (**E**) Summary of the data shown in (D). All data are presented as mean ± standard deviation of three different experiments. ns, no significant.

Previous studies show that C12 induces apoptosis in MEFs independent of Bak and Bax [25, 34], two pro-apoptotic Bcl-2 members required for MOMP in almost all apoptotic paradigms [35]. To clarify their role in C12-induced tumor cell apoptosis, human colon carcinoma HCT116 cell lines lacking Bak alone, Bax alone, or both Bak and Bax (Bak/Bax-DKO) were studied (Figure 4A). C12 caused roughly equivalent cell death and caspase-3/7 activation in all the HCT116 cell lines examined, indicating that deficiency of Bak and Bax in HCT116 cells did not influence their responses to C12 (Figure 4B–4C). Unlike C12, the therapeutic drug etoposide induced significant cell death and caspase-3/7 activation in wild-type (WT), Bax-KO and Bak-KO but not in Bak/Bax-DKO HCT116 cells (Supplementary Figure 7A–7B). Moreover, deficiency in Bak/Bax expression did not affect C12's effect to depolarize Δψ_mito_ (Figure 4D–4E) and release cytochrome c (Supplementary Figure 8), indicating Bak/Bax are not involved in C12-induced MOMP in tumor cells. These results demonstrate that C12-induced apoptotic signaling is distinct from that triggered by conventional anti-cancer drugs.

### The inhibitory effects of C12 on tumor growth are independent of Bak and Bax

**Figure 5:**
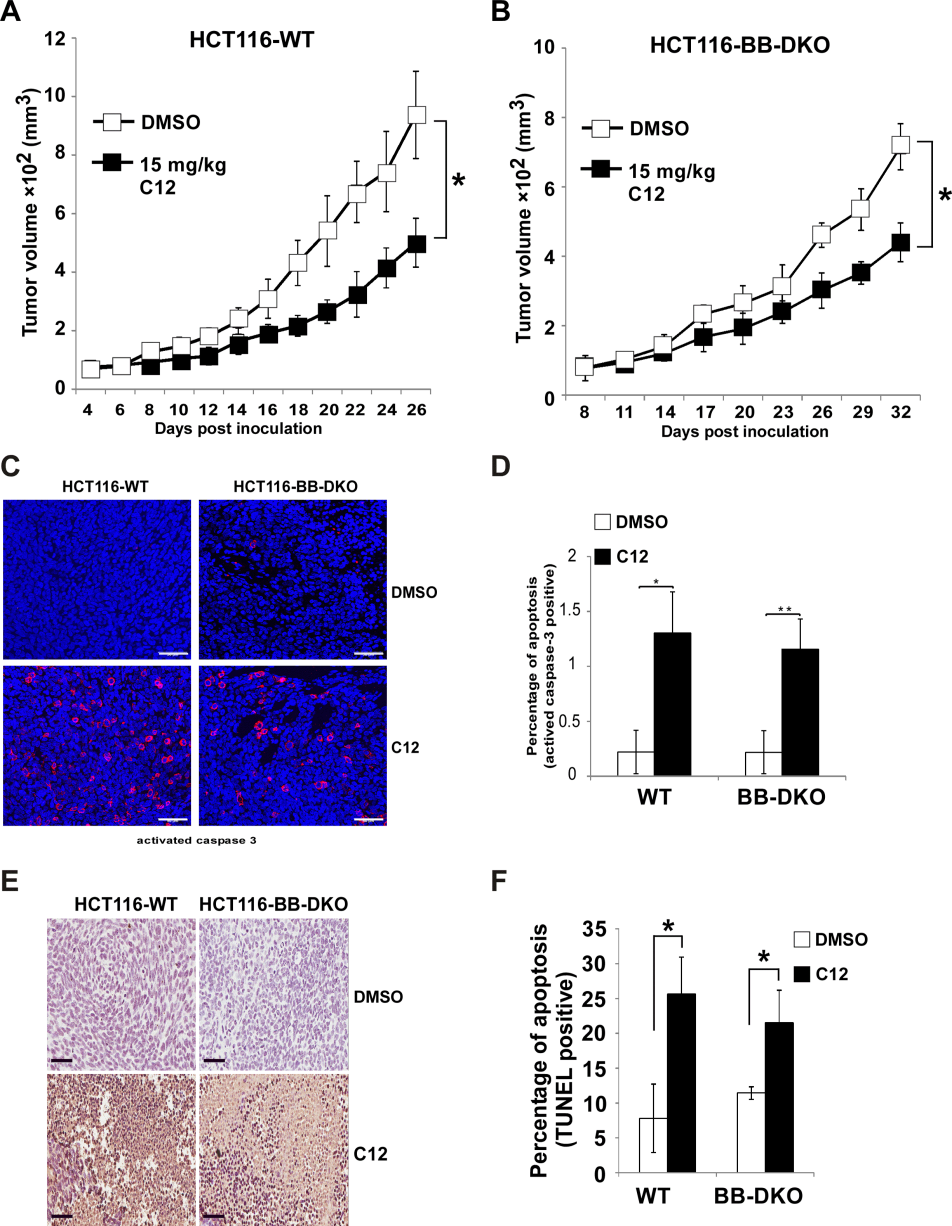
C12 inhibits xenografted tumor growth independent of Bak and Bax (**A**–**B**) Growth of HCT116-WT tumors (A) and HCT116-Bak/Bax-DKO tumors (B) in athymic nude mice treated with vehicle control or C12 (15 mg/kg/day). Data are mean ± standard deviation of tumor volumes of 8 animals in each group. (**C**) Apoptotic cells in tumor sections were detected by immunofluorescence staining of activated caspase-3. Representative images of tumor sections from DMSO- and C12-treated mice are shown. Scale bar, 50 μm. (**D**) The percentage of activated caspase-3 shown in (C) were quantified. Data are mean ± standard deviation of three independent tumor sections. Asterisk indicates *P* < 0.05 (*) or *P* < 0.01 (**) by student's unpaired *t* test (**E**) Typical TUNEL labeling images of tumor sections from vehicle control- and C12-treated mice are shown. Scale bar, 60 μm. (**F**) The percentage of TUNEL-positive cells in tumors of C12-treated mice is higher than that of control mice. All data are mean ± standard deviation of three independent tumor sections. Asterisk indicates *P* < 0.05 (*) by student's unpaired *t* test.

To further investigate whether the Bak/Bax-independent effect of C12 to trigger apoptosis *in vitro* also occurred *in vivo*, WT and Bak/Bax-DKO HCT116 cells were inoculated into athymic nude mice. C12 reduced the growth of established HCT116-WT tumors and HCT116-Bak/Bax-DKO tumors to a similar degree (Figure 5A–5B), indicating that Bak and Bax are not involved in anti-tumor activity of C12. Moreover, the level of activated caspase-3 and the percentage of TUNEL-positive cells were higher in C12-treated tumors than vehicle-treated tumors regardless of Bak and Bax expression levels (Figure 5C–5F), suggesting that C12 inhibits tumor growth probably through inducing Bak/Bax-independent apoptosis. Unlike C12, etoposide inhibited the growth of WT HCT116 tumors but not that of Bak/Bax-DKO HCT116 tumors (Supplementary Figure 7C–7D).

### BH-3 only proteins Noxa and Puma are not involved in C12-induced tumor cell apoptosis

**Figure 6:**
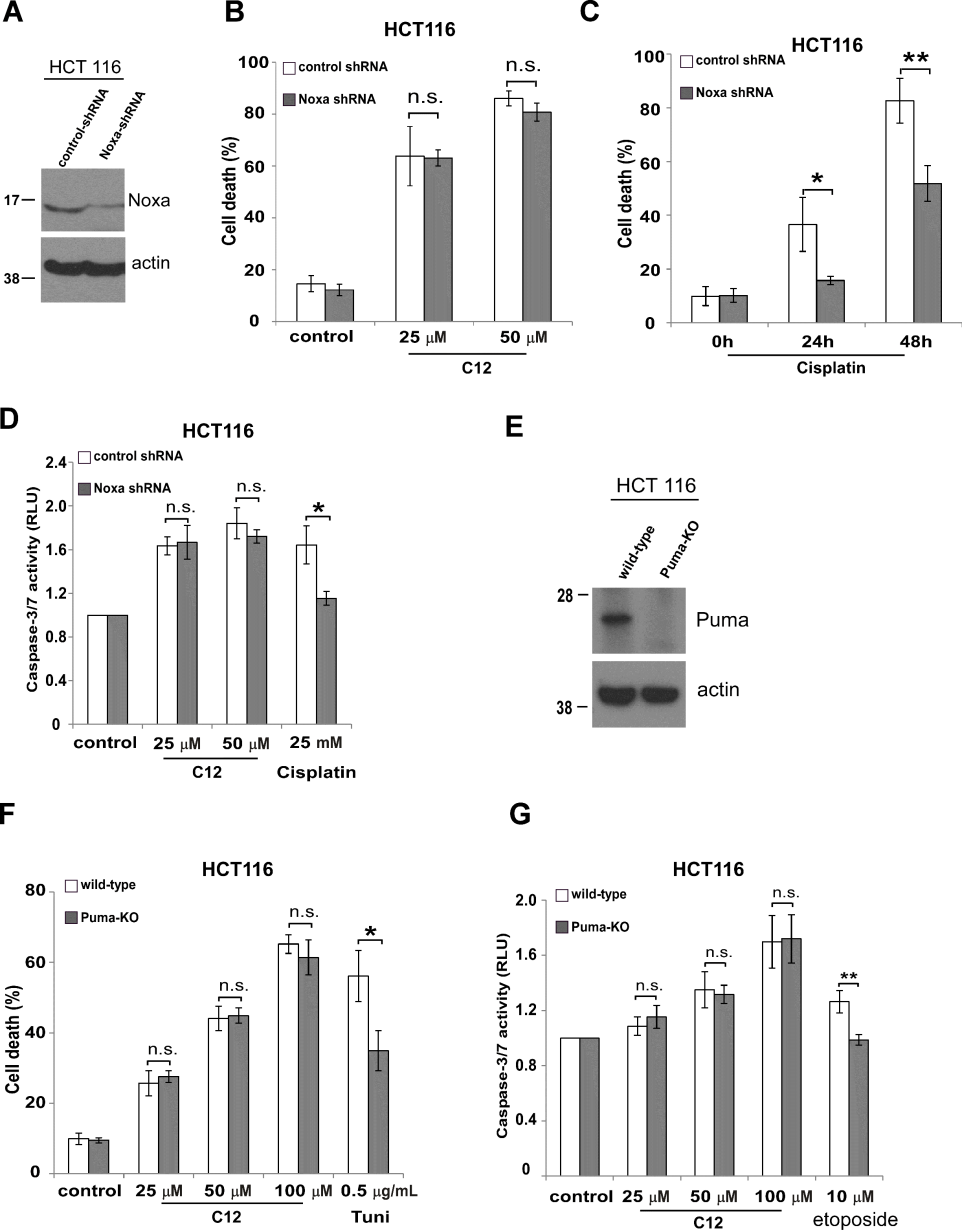
C12-induced tumor cell apoptosis is independent of BH-3 only proteins Noxa and Puma (**A**) Noxa expression was stably reduced in HCT116 cells by shRNA. (**B**) Following 24 hours treatment of C12, similar levels of cell death were detected in HCT116 cells expressing Noxa shRNA and their control counterparts. (**C**) Less apoptosis was detected in HCT116 cells with reduced Noxa expression upon treatment of cisplatin (50 μM). (**D**) Caspase-3/7activities were measured after 24 hour-incubation of C12 or cisplatin in the indicated HCT116 cells. (**E**) Puma expression in the indicated HCT116 cells was examined by western blot. (**F**) Cell death was measured after 24 hour-incubation of C12 or tunicamycin in Puma-deficient HCT116 cells or wild-type cells. (**G**) Caspase-3/7 activities were examined 24 hours after C12 or etoposide exposure. All data are presented as mean ± standard deviation of three different experiments. Asterisk indicates *P* < 0.05 (*) or *P* < 0.01 (**) by student's unpaired *t* test. ns, no significant.

BH3-only proteins are another class pro-apoptotic Bcl-2 proteins, which share sequence homology only in the BH3 domain, including Noxa and Puma [36]. To further elucidate whether BH3-only proteins are involved in C12 cytotoxicity in tumor cells, we investigated two HCT116 cell lines in which Noxa expression was stably reduced by shRNA (Figure 6A) or Puma expression was eliminated genetically (Figure 6E) [37, 38]. C12 caused similar levels of cell death and caspase-3/7 activation in HCT116 cell lines with reduced expression of Noxa or Puma compared with their corresponding counterparts expressing normal levels of Noxa or Puma (Figure 6B and 6F–6G). In contrast, less cytotoxicity was observed in HCT116 cells with decreased Noxa or Puma expression in response to conventional apoptosis stimuli (Figure 6C and 6F–6G). Overall, our results suggest that BH3-only proteins are not critical for C12-induced apoptotic signaling.

### PON2 expression was increased in human lung tumor tissues and oncogenically transformed HBE cells

**Figure 7:**
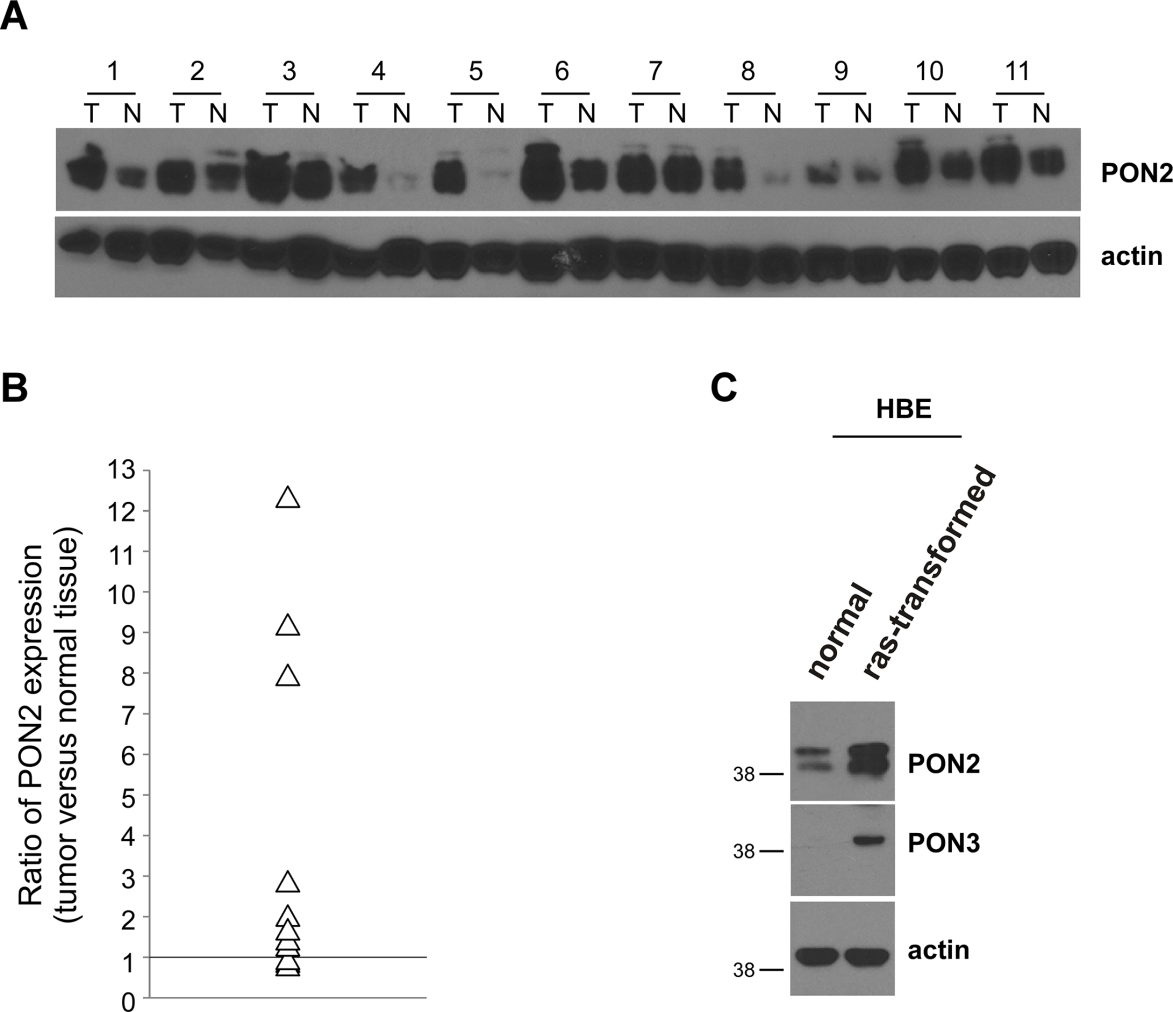
PON2 expression is enhanced in human lung tumor tissues and oncogenically transformed HBE cells (**A**) Expression of PON2 in NSCLC tissue specimens and corresponding adjacent normal tissues from 11 patients were evaluated by western blot. Samples 1–4, 6, 8, 9, 11 were from adenocarcinoma patients, whereas samples 5, 7, 10 were from squamous cell carcinoma patients. T, tumor; N, normal. (**B**) The intensities of bands in (A) were quantified using ImageJ software (NIH). To normalize loading variation, the relative levels of PON2 were calculated by dividing the PON2 value into the corresponding value for actin. The data were shown as a ratio of PON2 levels in a tumor tissue sample versus its corresponding normal tissue, and the value bigger than 1 indicates that PON2 expression is increased in tumor tissues. Differential expression of PON2 in tumor versus normal tissues is significant with the value of “P” smaller than 0.01 as calculated by student's paired *t* test. (**C**) The expression of PON2 and PON3 in primary HBE cells and their transformed counterparts was determined by western blot.

It has been shown that PON2 upregulation in some cancer cells, including lung cancer cell lines, enables cancer cells to become resistant to conventional therapeutic drugs [23]. To determine whether PON2 expression is enhanced in human lung cancer, we examined PON2 protein levels in tumor tissues of non small cell lung carcinoma (NSCLC) patients by western blot (Figure 7A). Among eleven samples from patients, we found that PON2 was overexpressed in eight of lung cancer tissues compared with corresponding adjacent normal tissues, whereas its expression was slightly decreased in three of them (Figure 7B). As Ras-transformed HBE displayed higher levels of apoptosis compared with their untransformed counterparts upon C12 treatment (Figure 1A–1B and Supplementary Figure 1), PON2 expression was also increased in transformed HBE cells (Figure 7C), providing more evidence that oncogenic transformation enhances PON2 expression.

### PON2 is involved in C12's cytotoxicity on tumor cells and inhibitory effects on tumor growth

**Figure 8:**
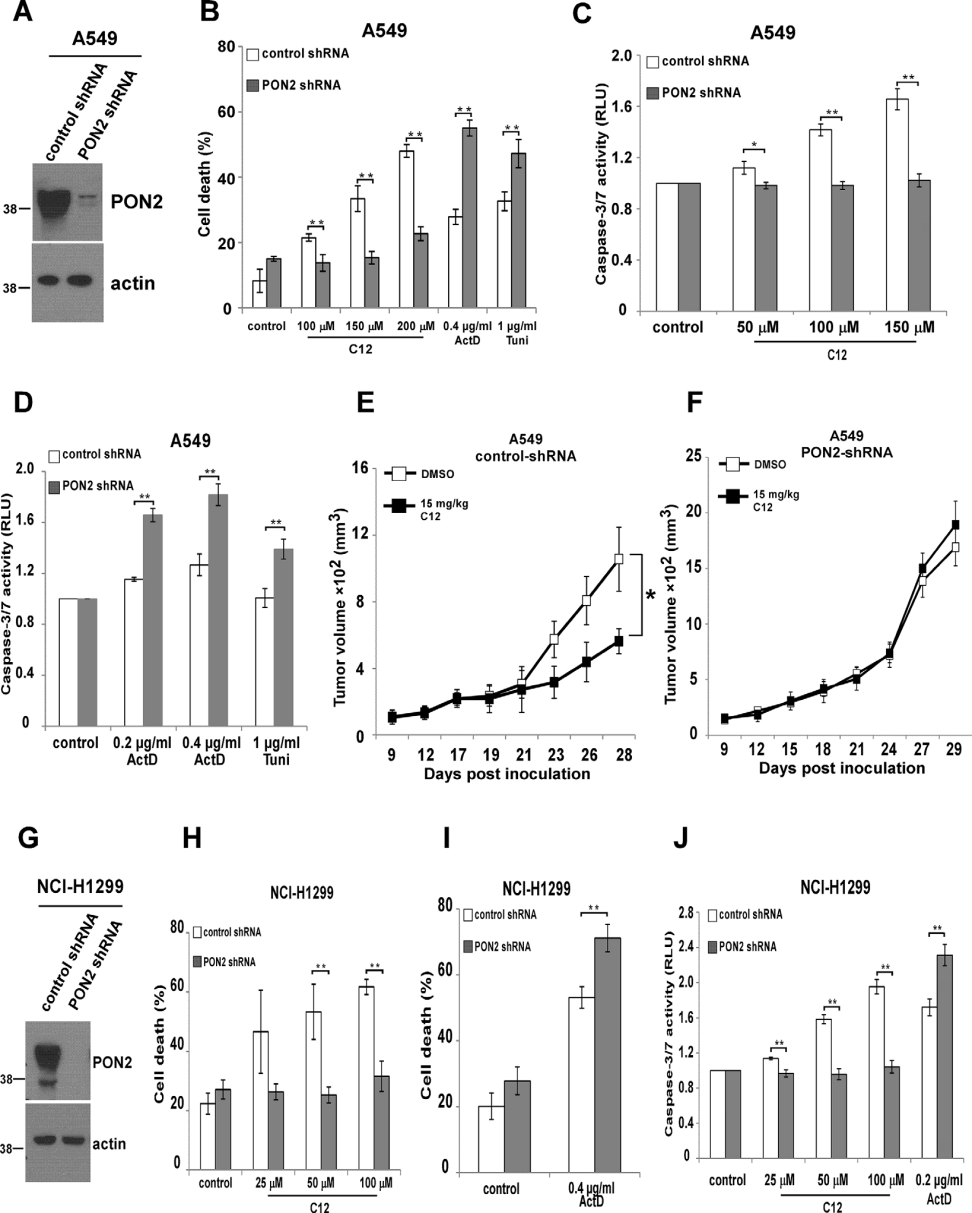
PON2 is required in C12's cytotoxicity on tumor cells and inhibitory effects on tumor growth (**A**) PON2 expression in A549 cells was stably reduced by shRNA. The expression levels of PON2 were determined by western blot. (**B**) C12 induced less apoptosis in A549 cells with reduced PON2 expression than in control vector cells, which is opposite to the effects of actinomycin D or tunicamycin. Cell death was assessed after 32 hour incubation. (**C**) Upon treatment with different doses of C12 for 32 hours, less caspase-3/7 activation was detected in cells with reduced PON2 expression than control vector cells. (**D**) Actinomycin D or tunicamycin induced more caspase-3/7 activation in A549 cells with higher PON2 expression following 48 hour treatment. (E-F) Growth of A549-vector tumors (**E**) and A549 tumors with reduced PON2 expression (**F**) in athymic nude mice treated with vehicle control or C12 (15 mg/kg/day). Data are mean ± standard deviation of tumor volumes of 7 animals in each group. Asterisks indicate *P* values of < 0.05 (*) by student's unpaired *t* test. (**G**) Stable reduction of PON2 expression in NCI-H1299 cells was evaluated by western blot. (**H**) C12 induced less cell death in NCI-H1299 cells with reduced PON2 following 24 hour treatment. (**I**) More cell death was detected in NCI-H1299 cells with lower PON2 expression following 24 hour exposure of actinomycin D. (**J**) Less apoptosis was detected in NCI-H1299 cells with reduced PON2 expression than control vector cells induced by C12, which is opposite to the effect of actinomycin D. Cell death was assessed after 24 hour incubation. All data shown are mean ± standard deviation of three independent experiments performed in triplicate. Asterisks indicate *P* values of < 0.05 (*) or < 0.01 (**) by student's unpaired *t* test.

In nontransformed MEF and HEK293T cells, overexpression of PON2 promotes cytotoxicity of C12 [25], but the role of endogenous PON2 in C12-evoked apoptotic signaling was unclear. To further investigate the mechanism of C12-triggered apoptosis, we studied the involvement of endogenous PON2 in C12 cytotoxicity in tumor cells. PON2 expression was stably reduced in human NSCLC cell lines A549 and NCI-H1299 using shRNA (Figure 8A and 8G). Upon treatment with C12, less cell death and caspase-3/7 activation were observed in A549 and NCI-H1299 cells lacking PON2 expression (Figure 8B–8D, 8H and 8J). In contrast, more cell death and caspase-3/7 activation were detected in PON2-deficient cells in response to the common apoptotic stimuli actinomycin D and tunicamycin (Figure 8B, 8D and 8I–8J), suggesting that PON2/C12 interaction induces a novel form of apoptosis distinct from that evoked by classical apoptotic stimuli. Importantly, C12 inhibited the growth of vector control A549 tumors but not the A549 tumors with reduced PON2 (Figure 8E–8F), further indicating that PON2 is vital for C12 cytotoxicity *in vivo*. In addition, similar levels of reduction in cell death and caspase-3/7 activation were observed in HCT116-WT and HCT116-Bak/Bax-DKO cells lacking PON2 expression (Supplementary Figure 9), demonstrating that Bak and Bax are not involved in PON2-mediated C12 cytotoxicity.

Previous studies indicate that PON2 reduces cellular oxidative damage and influences redox signaling and subsequent apoptotic pathways [23, 39, 40]. We investigated whether C12 modulated the antioxidant activities of PON2. Consistent with earlier findings, reducing PON2 expression increased intracellular reactive oxygen species (ROS) levels in A549 and NCI-H1299 cells (Supplementary Figure 10). However, C12 failed to influence ROS production regardless of PON2 expression levels, indicating that the interaction between C12 and PON2 does not modulate the antioxidant activities of PON2.

### PON3 expression recovers C12 cytotoxicity in PON2-deficient tumor cells

**Figure 9:**
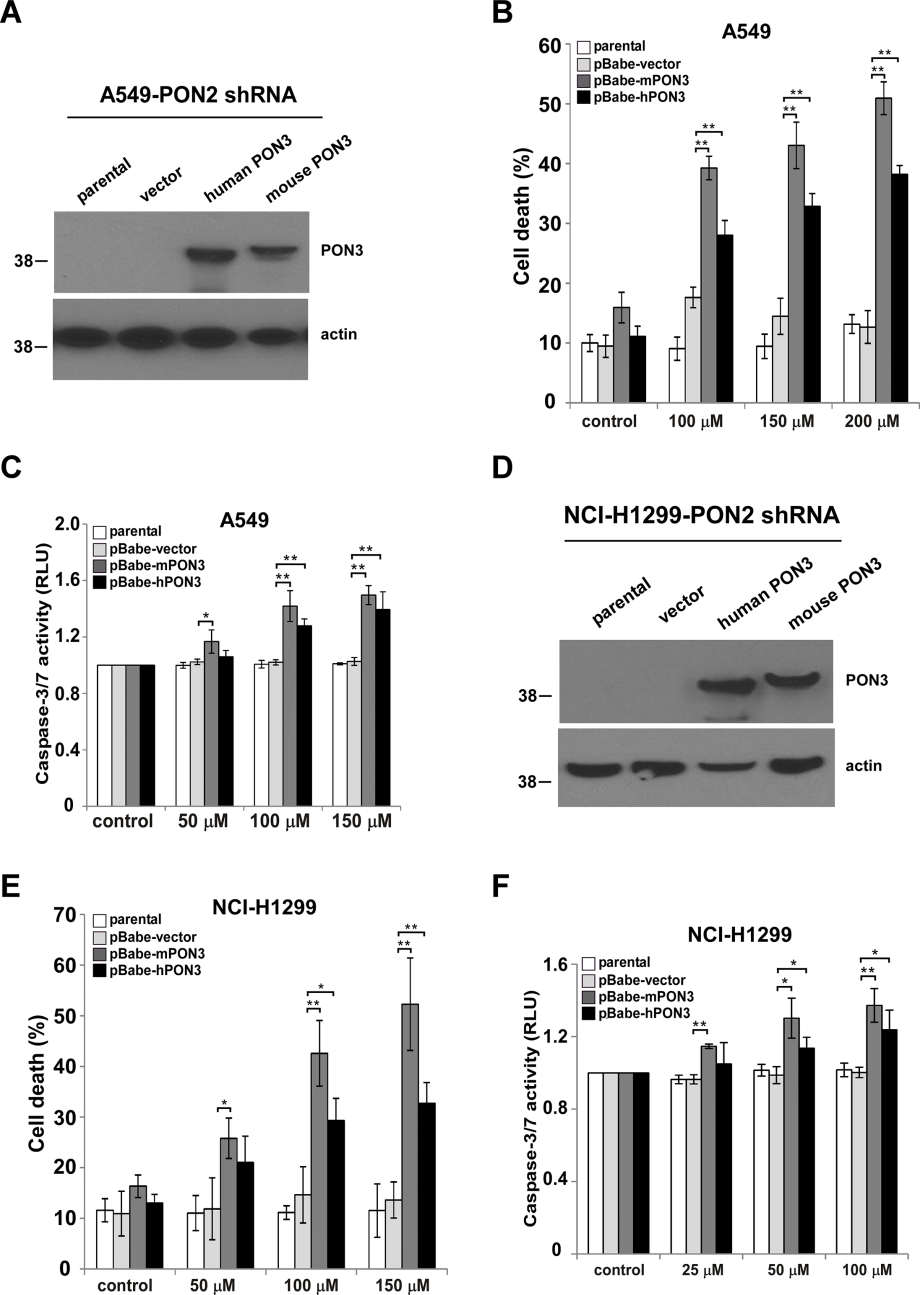
PON3 sensitizes tumor cells with reduced PON2 expression to C12 (**A**) Human or murine PON3 cDNA was stably overexpressed in A549 cells with reduced PON2 expression by retroviral infection. Expression levels of PON3 were determined by western blot. (**B**–**C**) After treating with different doses of C12 for 24 hours, more cell death (B) and caspase-3/7 activation (C) were detected in PON2-knockdown A549 cells with increased mouse and human PON3 expression comparing to vector control and parental cells. (**D**) Stable overexpression of human or murine PON3 in PON2-knockdown NCI-H1299 cells was examined by western blot. (**E**–**F**) C12 induced more cell death (E) and caspase-3/7 activation (F) in PON2-knockdown NCI-H1299 cells expressing mouse and human PON3 after 24 hours treatment. All data shown are mean ± standard deviation of three independent experiments performed in triplicate. Asterisks indicate *P* values of < 0.05 (*) or < 0.01 (**) by student's unpaired *t* test.

PON2 is known to function as a lactonase to cleave C12 [21, 41, 42]. Therefore, we reasoned that PON2 lactonase activity might be essential for C12 cytotoxicity on tumor cells. Since PON3 possesses overlapping enzymatic activities with PON2 to cleave C12 [43], we investigated whether PON3 could functionally compensate for the loss of PON2 and re-sensitize tumor cells to C12. To this purpose, the expression of human or murine PON3 was stably increased in PON2-deficient A549 and NCI-H1299 cells using retroviral infection. Western blot analysis showed that human PON3 and murine PON3 expression was significantly increased (Figure 9A and 9D). Overexpression of human or murine PON3 restored the sensitivity of PON2-deficient cells to C12, as measured by the levels of cell death and caspase-3/7 activation (Figure 9B–9C and 9E–9F). These data further validate the role of PON lactonase activity in C12-initiated apoptotic signaling in tumor cells.

## DISCUSSION

Despite growing evidence that the bacterial quorum-sensing molecule C12 induces apoptosis in various types of human tumor cells [5–8], the relevance of C12 cytotoxicity to tumor growth in animals was unknown. Importantly, knowledge about the mechanism of C12-evoked tumor cell apoptosis has been limited. Here, we present the evidence that C12 preferentially triggers transformed cell apoptosis *in vitro* and inhibits transplanted tumor growth *in vivo* as a single agent independent of both anti- and pro-apoptotic Bcl-2 proteins. Furthermore, selective cytotoxicity of C12 on lung tumor cells and its inhibitory effects on tumor growth are likely related to upregulated PON2 expression in tumor cells.

Many neoplastic cells show an increased ratio of anti-apoptotic to pro-apoptotic Bcl-2 proteins, which enables them to survive even under the conditions that would normally initiate apoptotic signaling [9, 44]. An emerging strategy for cancer therapy is to overcome the resistance to apoptosis caused by aberrant Bcl-2 signaling in tumor cells [45–47]. Recently, several small molecules triggering apoptosis independent of either pro- or anti-apoptotic Bcl-2 proteins have been identified as potential anti-tumor drugs. Among them, the pentacyclic triterpenoid betulinic acid induces Bak/Bax-independent MOMP and subsequent apoptosis [48]. Unlike C12, cytotoxic effects of betulinic acid are influenced by Bcl-2 overexpression and it is ineffective against epithelial tumors. Similarly, Bak/Bax is also nonessential in apoptotic signaling induced by chelerythrine [49], mitochondrial Kv1.3 channels inhibitors [50] or titanium dioxide (TiO_2_) [51]. The polyphenolic compound gossypol evokes Bak/Bax-independent apoptosis and inhibits Bcl-2-overexpressing tumor growth [52]. Furthermore, Bcl-2 expression fails to influence human tumor cell apoptosis induced by the antibiotic agent Tetrocarcin-A or protein complex HAMLET [16, 17]. Compared with those molecules, C12 is the first small molecule compound, to the best of our knowledge, inducing human tumor cell apoptosis *in vitro* as well as blocking tumor growth *in vivo* independent of both pro- and anti-apoptotic Bcl-2 proteins.

The apoptosis cascade induced by C12 in tumor cells is unique, evident by its rapid pro-apoptotic effects, such as depolarizing mitochondrial membrane potential within minutes (Figures 2 and 4), releasing cytochrome c into the cytosol within three hours (Supplementary Figures 6 and 8) and maximally activating caspases within four hours (Figure 1). These distinctive pro-apoptotic features of C12 have not been observed in any other apoptosis paradigms of cancer cells, which might be attributed to the ability of C12 or its derivatives generated in tumor cells to directly permeabilize mitochondria (within minutes) without the involvement of pro- and anti-apoptotic Bcl-2 proteins.

This study also shows that endogenous PON2 is essential for C12‘s cytotoxicity in human lung tumor cells and inhibitory effects on tumor growth (Figures 8–9), consistent with our previous observation of PON2 overexpression in nontransformed fibroblasts and HEK293T cells [25]. The ability of PON3 to restore the sensitivity of PON2-deficient tumor cells to C12 suggests that overlapping enzymatic activities of PON2 and PON3 mediate C12 killing activities. In addition, overexpressing wild-type but not lactonase-deficient PON2 in HEK293T cells sensitizes them to C12 [25]. These observations raise the possibility that a secondary, cytotoxic metabolite(s) of C12 generated by PON2 or PON3 is a more potent apoptosis inducer, which could be a carboxylic acid cleavage product of C12 or a molecule generated from its reaction with other molecules (e.g. phosphorylation). Future studies are warranted to identify such C12 metabolite(s).

It has been reported that lung tumor cell lines are resistant to conventional therapeutic drugs partially due to enhanced expression of PON2, which is thought to be associated with anti-oxidant activities of PON2 [23, 24]. Our data also show that PON2 expression is enhanced in lung cancer tissues from NSCLC patients and human bronchia/tracheal epithelial cells transformed by an oncogenic Ras allele (Figure 7). Moreover, C12's ability to kill NSCLC tumor cells *in vitro* and block tumor growth *in vivo* is mediated through PON2 (Figure 8). These results indicate that the inhibitory effects of C12 on lung tumor growth in both normal and immunodeficient nude mice (Figures 3 and 5) could be attributed to PON2 overexpression in tumors. Thus, it is conceivable that C12 or compounds derived from C12 could trigger rapid and Bcl-2 protein-independent apoptosis in lung tumors that are resistant to traditional chemotherapeutic drugs, whereas normal tissues are spared due to their lower PON2 expression.

As *Pseudomonas aeruginosa* produces C12 to control virulence factors, C12 alone could potentially promote infections, particularly in immune compromised patients [20]. Published information about the safety of C12 *in vivo* is limited except that C12 exerts acute influence in immune responses in mice. For instance, C12 reduces innate immune responses acutely via disrupting TLR4-dependent NF-kB signaling in mice [53]. In a murine model of dermal inflammation, intradermal injection of C12-loaded micelles leads to local immune cell infiltration within 24 hours [54]. In our studies, administration of C12 for extended periods of time does not appear to cause significant toxicity to either normal or immunodeficient athymic nude mice (Supplementary Figure 2), suggesting that long-term side effects of administrating C12 on animals might be limited.

Overall, our study reveals that C12 inhibits tumor growth in animals as a single agent, through inducing a unique Bcl-2 protein-independent and PON2-mediated apoptotic cascade in tumors. Therefore, C12 is an ideal candidate of a lead compound for novel therapeutic agents for cancer.

## MATERIALS AND METHODS

### Reagents

N-(3-oxododecanoyl)-homoserine lactone (C12), polybrene and actinomycin D were purchased from Sigma (St. Louis, MO). Propidium iodide (PI), CM-H2DCFDA (5-(and-6)-chloromethyl-2′,7′-dichlorodihydrofluorescein diacetate, acetyl ester), TMRE (tetramethylrhodamine, ethyl ester) and TOTO-3 were obtained from Invitrogen (Carlsbad, CA). Unless otherwise stated, all reagents were dissolved in dimethyl sulfoxide (DMSO). Dulbecco's Modified Eagle's Medium (DMEM), penicillin/streptomycin, trypsin, and L-glutamine were obtained from Mediatech (Manassas, VA), and fetal bovine serum was purchased from Gemini (Broderick, CA). Caspase-Glo assay 3/7 kit was purchased from Promega (Madison, WI). Antibodies (Abs) for western blot were anti-β-actin mAb (Sigma), anti-Bcl-2 mAb; anti-Bax pAb (Santa Cruz; Dallas, TX), anti-Bak pAb (Millipore; Billerica, MA), anti-PON2 pAb; anti-PON3 pAb; anti-Mcl-1 pAb (Abcam; Cambridge, MA), anti-Noxa pAb (Novus; Littleton, CO), anti-Puma pAb (ProSic; Poway, CA) peroxidase-conjugated goat anti-rabbit IgG (Thermo Fisher; Waltham, MA) and peroxidase-conjugated goat anti-mouse IgG (Thermo Fisher).

### Plasmids

The retroviral expression vector pBABE-IRES-mKate2 was generated by replacing EGFP cDNA in pBABE-IRES-EGFP with mKate2 cDNA. Human PON3 cDNA (Sino Biological; China) or murine PON3 cDNA (Origene; Rockville, MD) was cloned into pBABE-IRES-mKate2 to generate pBABE-hPON3-IRES-mKate2 or pBABE-mPON3-IRES-mKate2. pBABE-hBcl-2-IRES-EGFP was generated by cloning human Bcl-2 cDNA into pBABE-IRES-EGFP. pMIG-hMcl-1-IRES-EGFP and pMIG-hBcl-x_L_-IRES-EGFP was a gift from Dr. Levi Beverly (University of Louisville). The plasmid identities were validated by sequencing. Lentiviral human PON2 shRNA plasmid was purchased from Santa Cruz.

### Retrovirus and lentivirus production and cell culture

Retrovirus and lentivirus were generated as described previously [55]. HCT116 cells expressing different levels of Bak and Bax were obtained from Dr. Richard Youle (National Institutes of Health). HCT116 cells stably expressing Noxa shRNA or the control luciferase shRNA were described previously [37]. Puma-deficient HCT116 cells were acquired from Dr. Bert Vogelstein (John Hopkins University). NSCLC cell line A549 cells were obtained from ATCC, which were produced from adenocarcinomic alveolar basal epithelial cells of a lung cancer patient [56]. NCI-H1299 cells were also acquired from ATCC. A549 cells expressing Mcl-1 shRNA or control scramble shRNA were described previously [31]. A549 cells overexpressing Bcl-2, Bcl-x_L_ or Mcl-1 were produced by retroviral infection as described previously [55]. To generate NCI-H1299 or A549 cells with reduced PON2 expression or vector control, we infected cells using respective lentiviral supernatants with 10 μg/ml polybrene. Stable cell lines were obtained by culturing cells in the medium containing1.5 mg/ml puromycin. To express PON3 in NCI-H1299 or A549 cells with reduced PON2 expression, corresponding cells were infected with the retroviral supernatants containing 10 μg/ml of polybrene. NHBE cells were purchased from Lonza (Walkersville, MD). hT/LT/Ras HBE cells were obtained from Professor Barrett Rollins (Harvard Medical School). Lewis Lung Carcinoma cells, NCI-H1299 and A549 cells were cultured as described previously [55]. NHBE and hT/LT/Ras cells were grown in BEGM supplemented with SingleQuots (LONZA). HCT116 cells were grown as described previously [57]. Cells were all cultured in a 5% CO_2_ humidified incubator at 37°C.

### Cell death analysis and Chou-Talalay synergism assay

Cell viability of NCI-H1299 or A549 re-expressing PON3 cells was measured by TOTO^®^-3 Iodide using flow cytometry (FACSCalibur) as described previously [51]. Cell viability in other studies was determined by propidium iodide exclusion using flow cytometry as described previously [55]. The synergistic effect of C12 and ABT-737 on cell viability was evaluated by Chou-Talalay median dose effect assay. IC_50_ of C12 and ABT-737 on NCI-H1299 or A549 was first determined. Various combinations of C12 and ABT-737 at a constant ratio above or below their IC_50_ values were added to cells. Following incubation for the indicated time, cell viability was evaluated and Fa (fractional activity) was determined as the ratio between the cell death levels of drug-treated cells and those of untreated control cells. Combination index (CI) was calculated by the CompuSyn software (Biosoft; Cambridge, UK).

### Caspase-3/7 activity and TUNEL labeling

Caspase-3/7 activities were measured using a Caspase-Glo assay kit (Promega; Madison, WI) as described previously [58]. The proluminescent substrate containing the amino acid sequence Asp-Glu-Val-Asp (DEVD) is cleaved by activated caspase-3/7, leading to the release of a luciferase substrate (aminoluciferin) and the generation of luminescent signal. Data were presented as relative luminescent units (RLU) which were normalized to the corresponding values of control cells as an indicator of caspase-3/7 activities. TUNEL labeling was carried out by the Pathology Research Services Laboratory at University of Washington. The slides were scanned by a ScanScope CS digital slide scanner (Aperio; Vista, CA) and analyzed by ImageJ software (NIH) as described previously [46].

### Measuring Δψ_mito_ using imaging microscopy

Cells were incubated with 10 μM Δψ_mito_ probe JC1 in growth media for 10 minutes at room temperature followed by three washes with Ringer's solution. Dye-loaded cells were mounted onto a chamber on the stage of a Nikon Diaphot inverted microscope with a Fluor 20x objective (0.75 NA) at room temperature. Treatments were made by diluting stock solutions into Ringer's solution at the indicated concentrations. Fluorescence imaging measurements of Δψ_mito_ were performed as reported previously [25, 34]. Briefly, a charge coupled device camera collected JC-1 emission images using filter wheels (Lambda-10, Sutter Instruments; Novato, CA). Images were corrected for background (region without cells). Quantitative data are reported as JC1 fluorescence ratios normalized to minimal JC1 ratios obtained at the start of the experiment and maximal JC1 ratios obtained after 5 μM FCCP treatment: Normalized JC1 Ratio = (Ratio at a given time − minimal Ratio) / (maximal ratio − minimal Ratio) *100%. Normalized JC1 ratios at plateau (before addition of FCCP) from different experiments have been averaged for summary charts.

### Mitochondrial morphology

To image mitochondrial morphology and membrane potential using confocal microscopy, cells were transfected with mitochondrial matrix-targeted GFP plasmid (Clontech; Madison, WI) using an Amaxa nucleofector device (Lonza; Allendale, NJ), seeded onto 35 mm glass coverslips and placed in a tissue culture incubator. After 16–24 hours coverslips were mounted in a recording chamber positioned on the stage of an inverted microscope (IX71; Olympus America Inc., Center Valley, PA). Cells were visualized using a PlanApo 60x, 1.42 NA oil immersion objective and confocal time series images acquired using a VT-Infinity 3 (VisiTech International; Sunderland, UK). The chamber was perfused with Hank's Balanced Salt Solution containing 20 nM mitochondrial membrane potential probe tetramethylrhodamine, ethyl ester (TMRE) at room temperature. After a 15 minute equilibration period, GFP and TMRE were alternately excited using the 488 and 568 nm lines respectively of a Krypon-Argon laser. The emitted fluorescence was filtered using a dual bandpass filter set (VisiTech International) and collected and using HCImage software (Hamamatsu Corporation; Sewickley, PA). Mitochondrial morphology was analyzed using ImageJ to determine the mean area/perimeter ratio and inverse circularity as the measurement of interconnectivity and elongation respectively, as described previously [59].

### Immunofluorescence microscopy

For immunofluorescence staining of A549 and HCT116 cells, cells plated 24 hours earlier were incubated for 3 hours with either vehicle (DMSO) or 50 μM C12 in Ringer's solution. The immunofluorescence staining of cytochrome c was carried out as described previously [55]. Images were captured using a Nikon Eclipse Ti confocal microscope with a 40x CFI Plan Fluor objective (NA 0.6). To minimize variability, the same microscope settings were used for all experiments.

For immunofluorescence staining of tumor sections, tumor sections (5 μm) were treated with antigen retrieval procedure by boiling in 10% Triton x-100, then slowly cooled down at room temperature. After incubating with the blocking buffer (1× PBS, 0.2% Triton X-100, 5% goat serum), the slides were incubated with antibodies against activated caspase-3 (Cell signaling) overnight at 4°C. Following three 10-minute washes, slides were incubated with goat anti-rabbit IgG (Alexafluor-568, Invitrogen) for 1 hour. The fluorescence was visualized by confocal microscopy using a 40x CFI Plan Fluor objective (NA 0.6) and was analyzed using ImageJ software (NIH) as described previously [60].

### *In vivo* animal studies

For C12 toxicity studies, DMSO or C12 (25 mg/kg/day) was administered intraperitoneally each day in 8 week-old C57BL/6 mice or in 6 week-old athymic nude mice. All organs were collected and weighted at sacrifice.

For transplanted tumors in C57BL/6 mice, eight-week old C57BL/6 female mice (Jackson Laboratories; Bar Harbor, ME) were inoculated subcutaneously (s.c.) with 1×10^6^ Lewis Lung Carcinoma cells on the right flank. Tumors were measured daily with dull edged Vernier calipers (V = L × W^2^/^2^). After tumor size reached around 100 mm^3^, animals with size-matched tumors were divided into control group and C12 group. DMSO or C12 was administered intraperitoneally each day. At the end of the experiments, tumors were excised for apoptosis evaluation. For the study of transplanted human tumors, female athymic nude mice (5–7 weeks) were purchased from Harlan Laboratories (Indianapolis, IN). Wild-type HCT116 cells and Bak/Bax DKO HCT116 cells (3.0 × 10^7^/ml), wild-type A549 cells and Bcl-2-overexpressing A549 cells (3.0 × 10^7^/ml), A549-control-shRNA cells and A549-PON2-shRNA cells (3.0 × 10^7^/ml) were resuspended and mixed 1:1 (vol/vol) with Matrigel (BD Biosciences), then 0.1 ml mixture of cells and Matrigel was injected s.c. on the right flank of mice. All animals with size-matched tumors were divided into two groups (control and C12) when the tumor size reached around 100 mm^3^. For HCT116 and Bak/Bax DKO HCT116 tumors, DMSO or C12 was administered intraperitoneally each day. DMSO or etoposide (40 mg/kg/day) was administered intraperitoneally once every other day. For wild-type A549 tumors, animals were treated with C12 for 6 times/week. For Bcl-2 over-expressing A549 cells tumors, DMSO or C12 was administered with C12 each day. For A549-control-shRNA and A549-PON2-shRNA tumors, DMSO or C12 was administered twice every three days. At the end of the experiments, tumors were excised for apoptosis evaluation.

### Lung tumor tissues from patients

Tumor tissues along with corresponding adjacent normal tissues from non small cell lung carcinoma (NSCLC) patients were acquired from the James Graham Brown Cancer Center Bio-Repository at University of Louisville following an approved IRB protocol. Frozen tissue samples were resuspended in tissue protein extraction reagent (Thermo Fisher) supplemented with protease inhibitors (Complete, Roche; Indianapolis, IN) and phosphatase inhibitors (PhosSTOP, Roche). Following homogenization and centrifugation, protein concentration was determined by BCA assay (Thermo Fisher). Thirty micrograms of total protein was electrophoresed in 4–12% Bis/Tris gels (Bio-Rad; Hercules, CA). PON2 was detected by western blot as described previously [46].

### ROS measurement

A549 or NCI-H1299 cells were cultured in 96-well white-walled plates (7.5 × 10^3^ per well) for 24 hours. Cells were subsequently treated with DMSO or 100 μM CM-H2DCFDA in growth medium for 1 hour at 37°C. Growth medium was then removed and cells were washed twice with Ringer's Buffer. DMSO or 100 μM C12 in Ringer's Buffer was added to cells. The fluorescence signal (Ex/Em = 492 nm/520 nm) was measured kinetically at 37°C using the Gemini EM microplate spectrofluormeter (Molecular Devices; Sunnyvale, CA). Data was plotted as relative fluorescence units (RFUs) versus time and the slope was determined (RFU/min) as an indicator of intracellular ROS level.

### Statistical analysis

Results are presented as mean ± standard deviation of at least three independent experiments. Statistical analysis was performed using Student's two tail *t*-test. A *p* value < 0.05 was considered significant.

## SUPPLEMENTARY MATERIALS FIGURES AND VIDEOS










