# Supplementary material for: N-(3-oxo-acyl) homoserine lactone inhibits tumor growth independent of Bcl-2 proteins
Source: Oncotarget. 2016 Jan 7;7(5):5924–42. doi: 10.18632/oncotarget.6827 (PMC4868731; doi:10.18632/oncotarget.6827)
Supplement: Supplementary file 1 [file oncotarget-07-5924-s001.pdf]

# N-(3-oxo-acyl)homoserine lactone inhibits tumor growth independent of Bcl-2 proteins

## Supplementary Materials

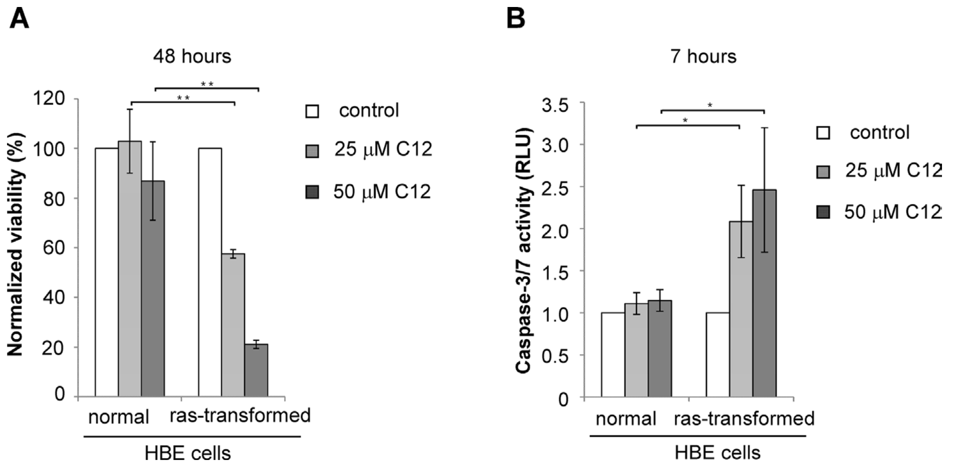


**Supplementary Figure S1: Cytotoxicity of C12 is influenced by oncogenic transformation.** (**A**) C12 induced more cell death in transformed HBE cells than their primary counterparts following 48 hour exposure. (**B**) More caspase-3/7 activation was detected in transformed HBE cells than primary HBE after the treatment of C12 for 7 hour. All data are the mean ± standard deviation of three independent experiments. Asterisk indicates $P < 0.05$ (*) or $P < 0.01$ (**) by student's unpaired *t* test.

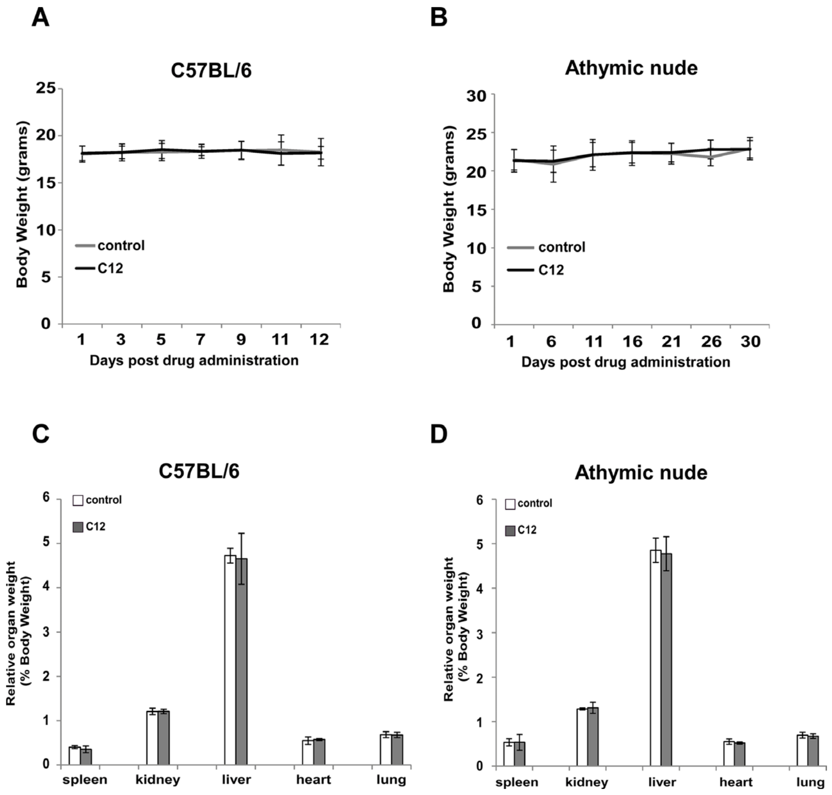


**Supplementary Figure S2: Administrating C12 to mice did not affect body weights and organ weights.** (**A–B**) DMSO or C12 (25 mg/kg/day) was administered intraperitoneally to C57BL/6 mice (A) or athymic nude mice (B) daily. Body weights of mice were measured. Data are mean ± standard deviation of body weights of 5 animals in each group. (**C–D**) Effects of C12 on organ weights in C57BL/6 mice (C) and athymic nude mice (D) were evaluated. Spleen, kidney, liver, heart and lung of the indicated mice were weighted at sacrifice. Each organ weight was normalized as the percentage of corresponding body weight. Data are mean ± standard deviation of organ weights of 5 animals in each group.

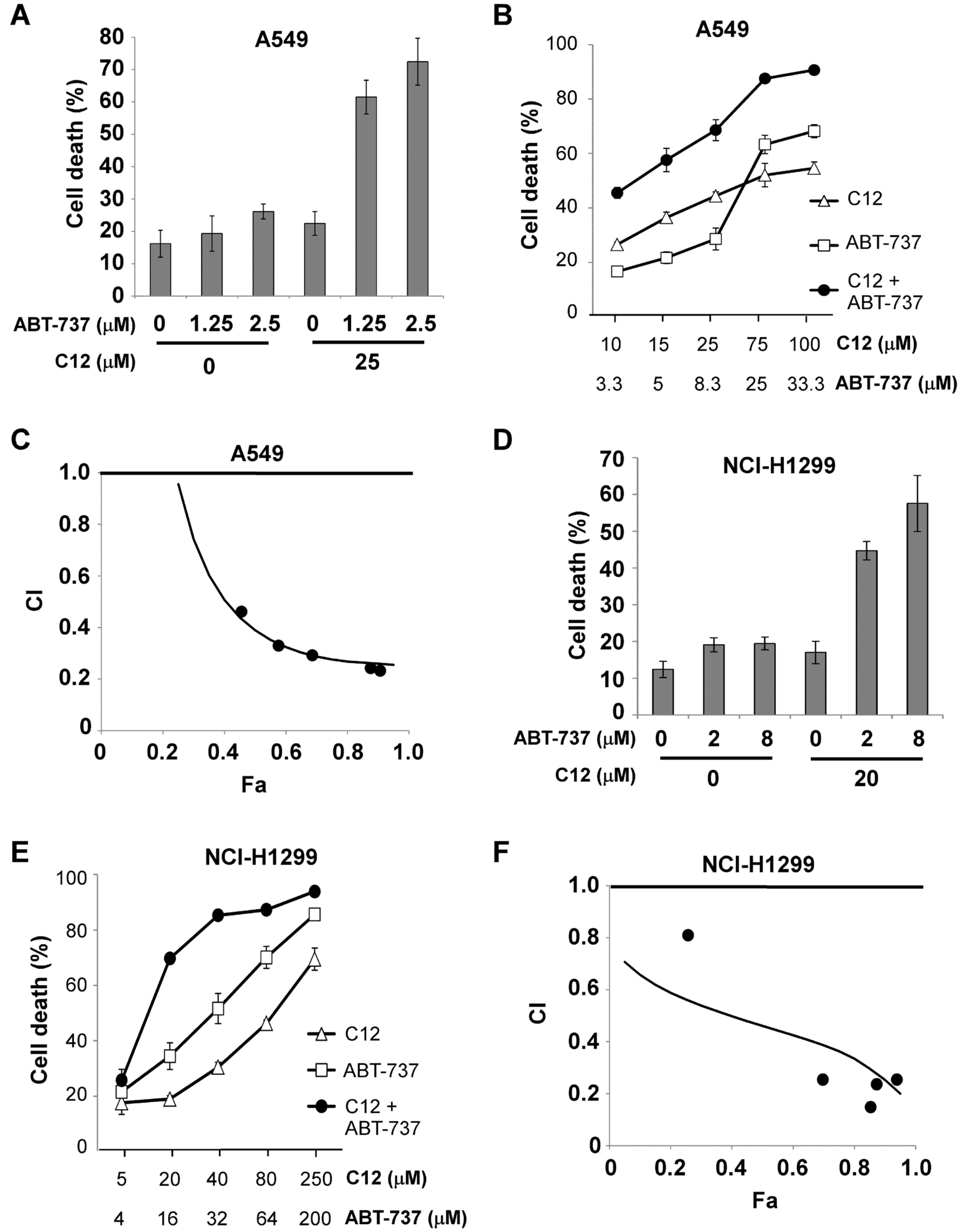


**Supplementary Figure S3: C12 functions synergistically with ABT-737 to induce tumor cell death.** (**A**) C12 sensitized A549 cells to ABT-737 treatment. Upon treatment with different combinations of C12 and ABT-737 for 48 hours, the percentage of cell death of A549 cells was measured. The data depict mean ± standard deviation of three independent experiments. (**B**) A549 cells were treated with the indicated combination of C12 (10-100 μM) and ABT-737 (3.3-33.3 μM) for 48 hours, and cell death was measured. The data represent mean ± standard deviation of triplicate experiments. The experiments were performed independently three times. (**C**) The data shown in (B) was analyzed as described in MATERIALS AND METHODS. The values of combination index (CI) and fractional activity (Fa) were calculated. (**D**) NCI-H1299 cells were treated with different combinations of C12 and ABT-737 for 48 hours, and cell viability was measured. The data are mean ± standard deviation of three independent experiments. (**E**) NCI-H1299 cells were cultured with the fixed combination of C12 (5-250 μM) and ABT-737 (4-200 μM) for 48 hours, and cell death was determined. The data are mean ± standard deviation of triplicate experiments. The experiments were performed independently three times. (**F**) The data shown in (E) was evaluated as described in MATERIALS AND METHODS and CI values were computed.

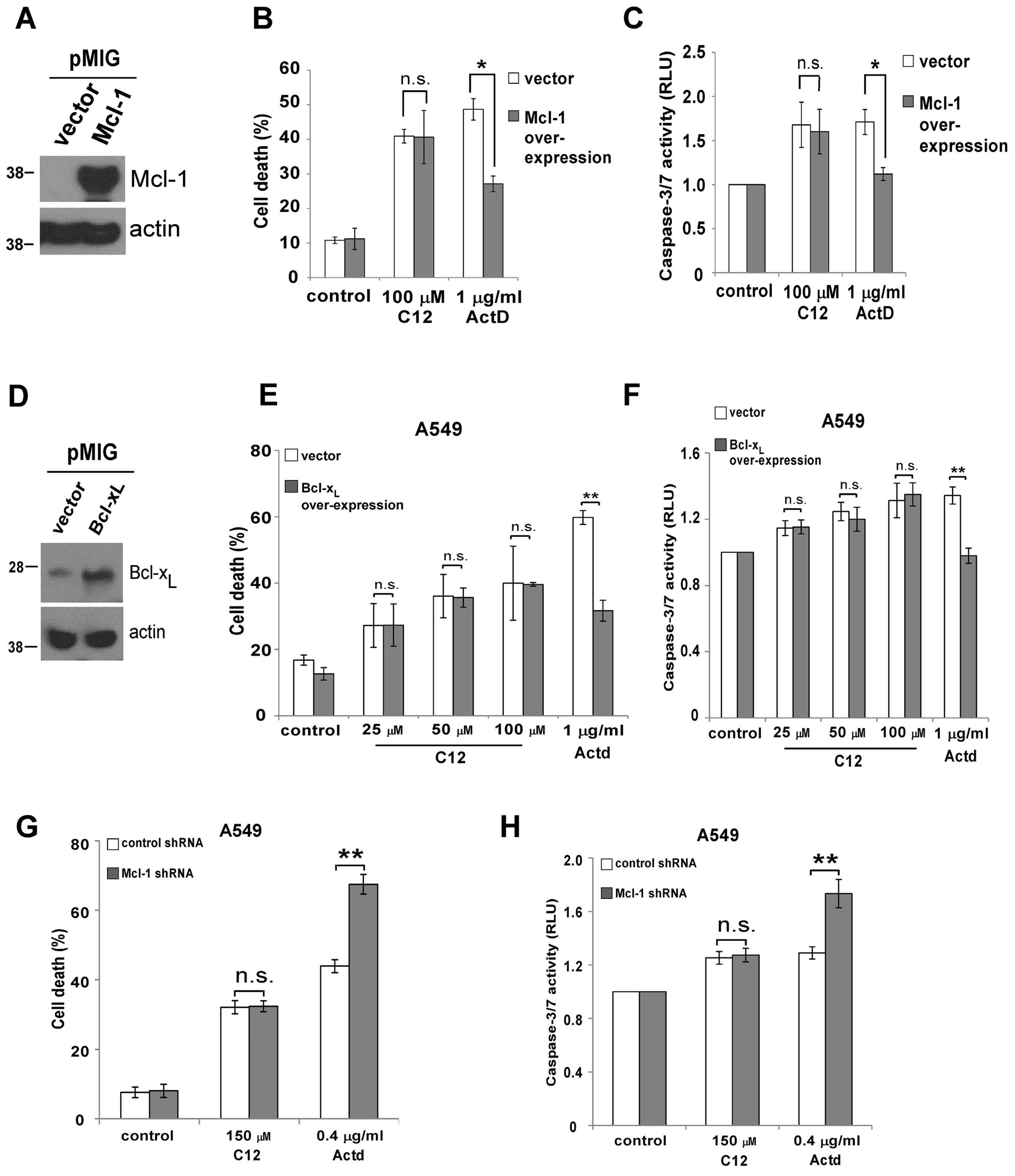


**Supplementary Figure S4: Anti-apoptotic Bcl-2 protein Mcl-1 and Bcl-$x_L$ does not affect C12 cytotoxicity.** (**A**) The expression levels of Mcl-1 in the indicated A549 cells were determined by western blot. (**B**) Cell viability of the indicated A549 cells with or without Mcl-1 over-expression was measured 48 hours after the treatment. (**C**) Caspase-3/7 activities were determined after exposure to C12 for 2 hours and exposure to actinomycin D for 24 hours. (**D**) Bcl-$x_L$ were stably overexpressed in A549 cells by retroviral infection. (**E**) Cell death was measured after 48 hour-incubation of C12 or actinomycin D (1 µg/ml) in A549 cells without or with over-expressed Bcl-$x_L$. (**F**) Caspase-3/7 activities were examined 32 hours after C12 or actinomycin D exposure. (**G**) After treating A549 cells expressing Mcl-1 shRNA or control shRNA with C12 or actinomycin D for 72 hours, cell death was measured. (**H**) Caspase-3/7 activities were measured after 32 hour-incubation of C12 or actinomycin D in the indicated A549 cells. All data are presented as mean ± standard deviation of three different experiments that were carried out in triplicate. Asterisk indicates $P < 0.05$ (*) or $P < 0.01$ (**) by student's unpaired $t$ test. ns, no significant.

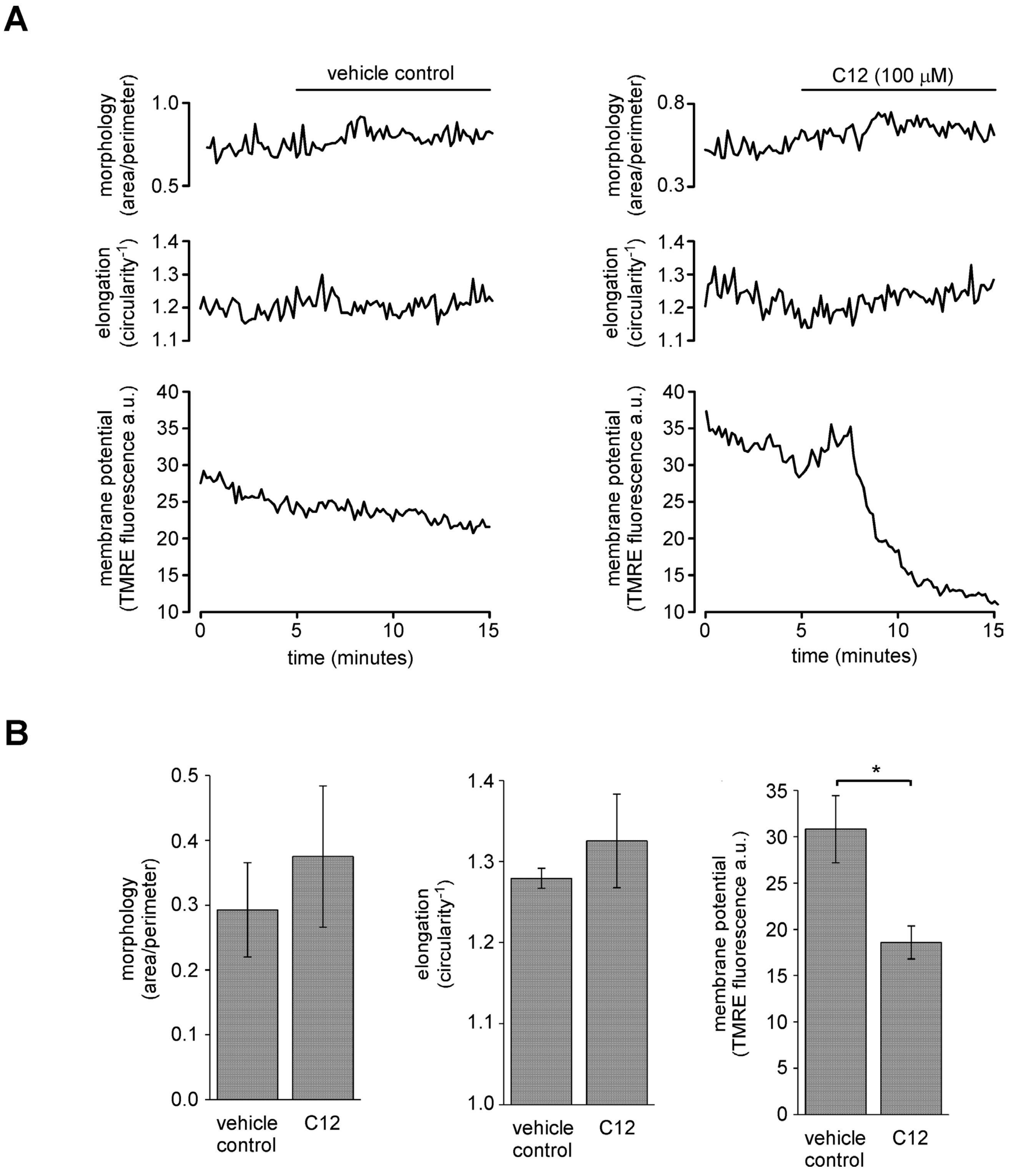


**Supplementary Figure S5: C12 depolarizes mitochondrial membrane potential without affecting mitochondrial morphology.** (**A**) Time-lapse confocal microscopy on single A549 cells was carried out in the presence of the vehicle control DMSO or 100 μM C12. Fluorescent signals of mitochondrial matrix-targeted GFP and TMRE were recorded simultaneously to evaluate mitochondrial morphology and membrane potential respectively. Typical traces of mitochondrial area/perimeter ratio, inverse circularity, and membrane potential of a single cell were shown. (**B**) Summary data (mean ± SEM) pooled from 3 independent coverslips run on two separate days depicting morphology and membrane potential at time 0 and after 10 minutes exposure to vehicle control or C12. Asterisk indicates $P < 0.05$ (*) by student's paired $t$ test.

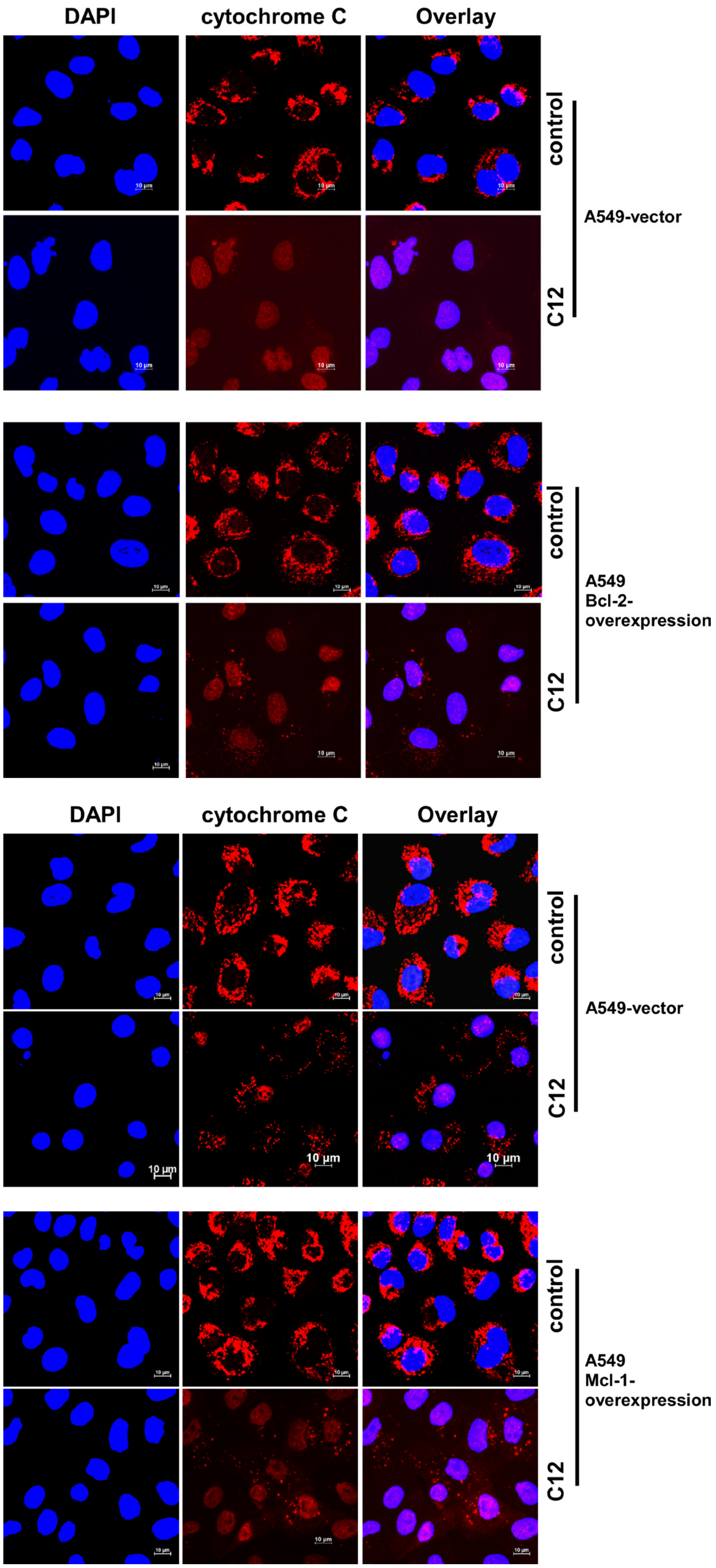


**Supplementary Figure S6: C12-induced cytochrome c release in tumor cells is independent of anti-apoptotic Bcl-2 proteins.**(**A**) Cytochrome c is released from mitochondria in both A549-vector and A549-Bcl-2 over-expressing cells upon C12 exposure. Representative confocal images of A549 cells treated with either DMSO (control) or 100 µM C12 for 3 hours. Cytochrome c is shown in red, and DAPI is shown in blue. Following C12 treatment, staining of cytochrome c became diffuse. Scale bar, 10 µm. (**B**) After treating with 100 µM C12 of 3 hours, cytochrome c was released from mitochondria in both A549-vector and A549-Mcl-1 over-expressing cells. Representative images acquired by confocal microscopy are shown. Scale bar, 10 µm.

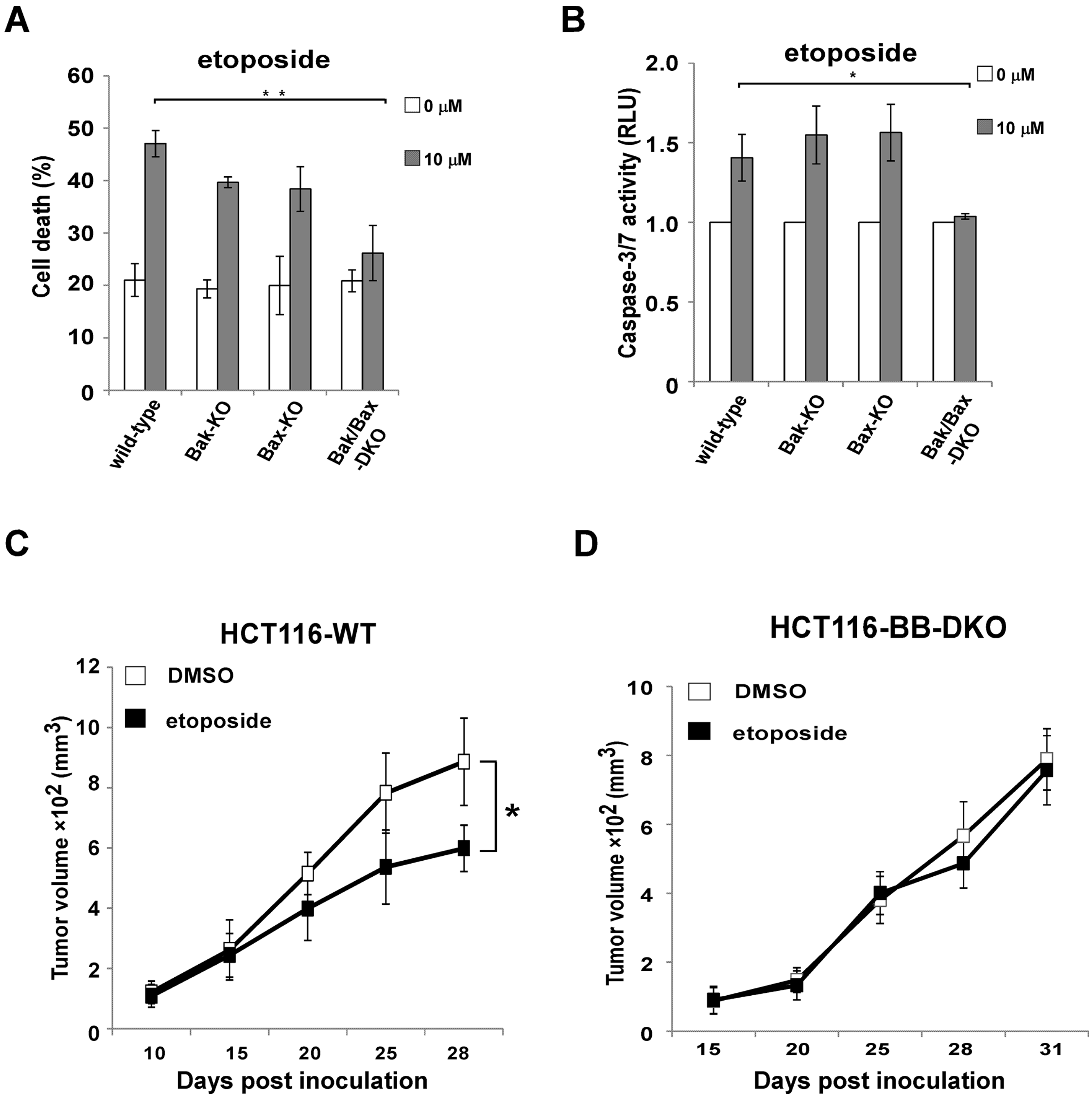


**Supplementary Figure S7: Etoposide induces tumor cell death and inhibits xenografted tumor growth dependent of Bak and Bax.** (**A**) Etoposide induced significant cell death in WT, Bak-KO and Bax-KO HCT116 cells but not in their Bak/Bax-DKO counterparts after 48 hours of treatment. (**B**) Caspase-3/7 activities were examined 24 hours after etoposide exposure. All the data are presented as mean ± standard deviation of three different experiments. Asterisk indicates $P < 0.05$ (*) or $P < 0.01$ (**) by student's unpaired *t* test. (**C–D**) Growth of HCT116-WT tumors (C) and HCT116-Bak/Bax-DKO tumors (D) in athymic nude mice treated with the vehicle control or etoposide. Data are mean ± standard deviation of tumor volumes of 5 animals in each group.

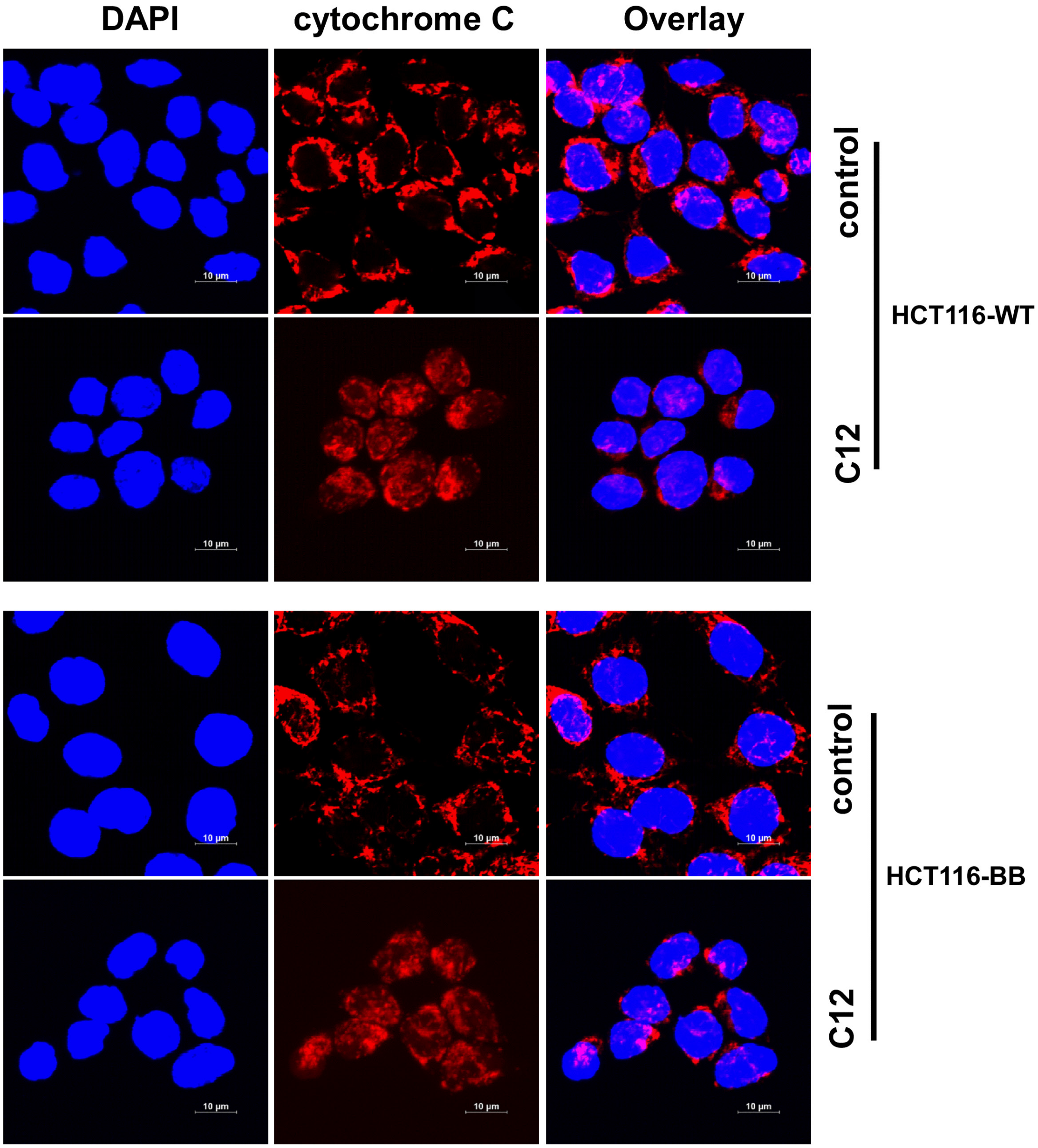


**Supplementary Figure S8: C12 triggered cytochrome c release in tumor cells independent of Bak and Bax.** (A–B) Cytochrome c was released from mitochondria in both HCT116-WT (A) and HCT116-Bak/Bax-DKO (B) cells upon treating with 100 µM C12 of 3 hours. Typical confocal images are shown. Cytochrome c is shown in red, and DAPI is shown in blue. Scale bar, 10 µm.

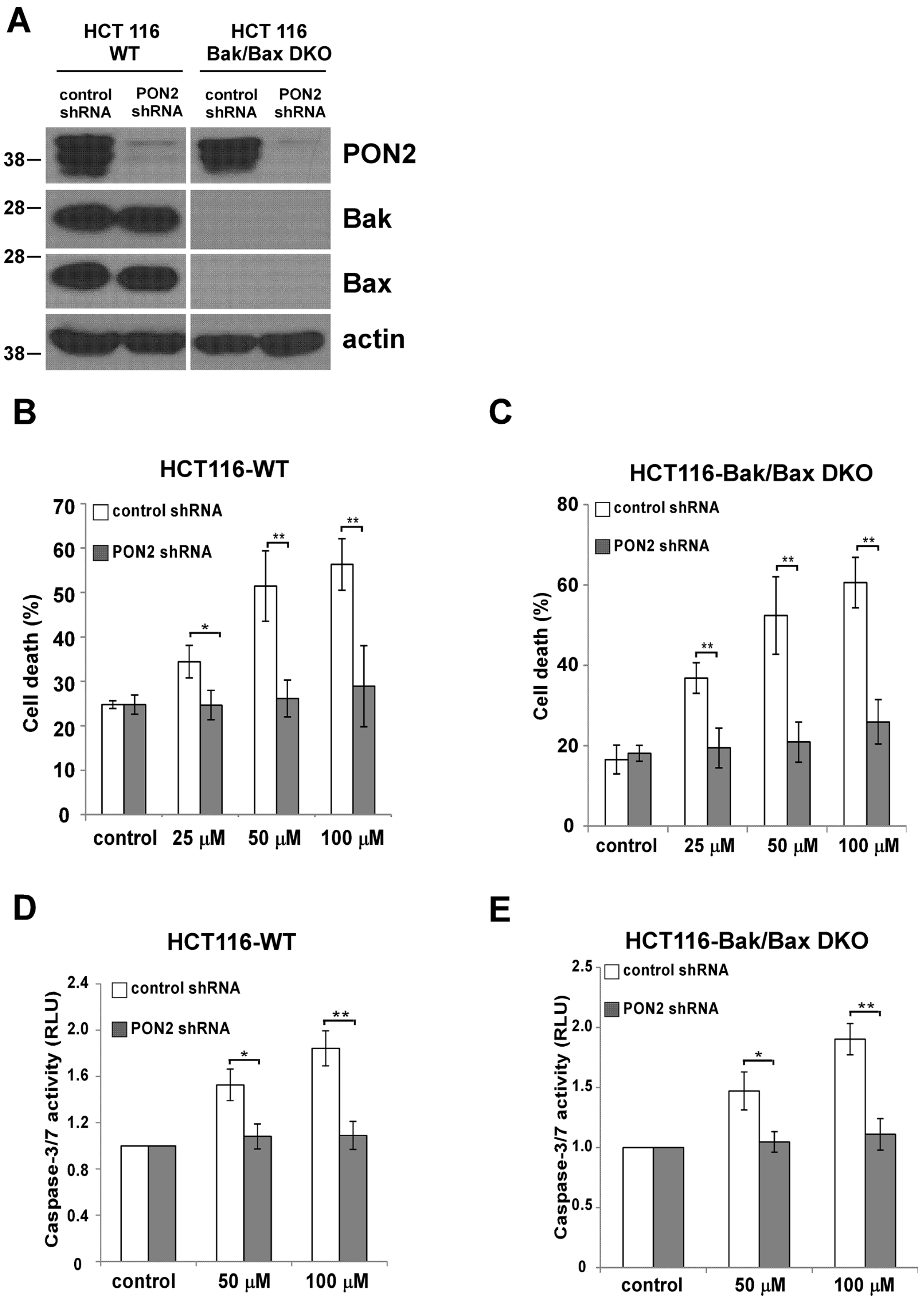


**Supplementary Figure S9: The involvement of PON2 in C12 cytotoxicity on tumor cells is independent of Bak and Bax.** (**A**) PON2 expression in HCT116-WT and HCT116-Bak/Bax-DKO cells was stably reduced by shRNA. The expression levels of PON2, Bak and Bax were determined by western blot. (B-C) C12 induced less cell death in HCT116-WT cells (**B**) and HCT116-Bak/Bax-DKO (**C**) with reduced PON2 expression than in control vector cells. Cell death was assessed after 24 hour incubation. (D-E) Upon treatment with different doses of C12 for 24 hours, less caspase-3/7 activation was detected in HCT116-WT cells (**D**) and HCT116-Bak/Bax-DKO (**E**) in cells with reduced PON2 expression than control vector cells. All data shown are mean ± standard deviation of three independent experiments performed in triplicate. Asterisks indicate $P$ values of < 0.05 (*) or < 0.01 (**) by Student's unpaired $t$ test.

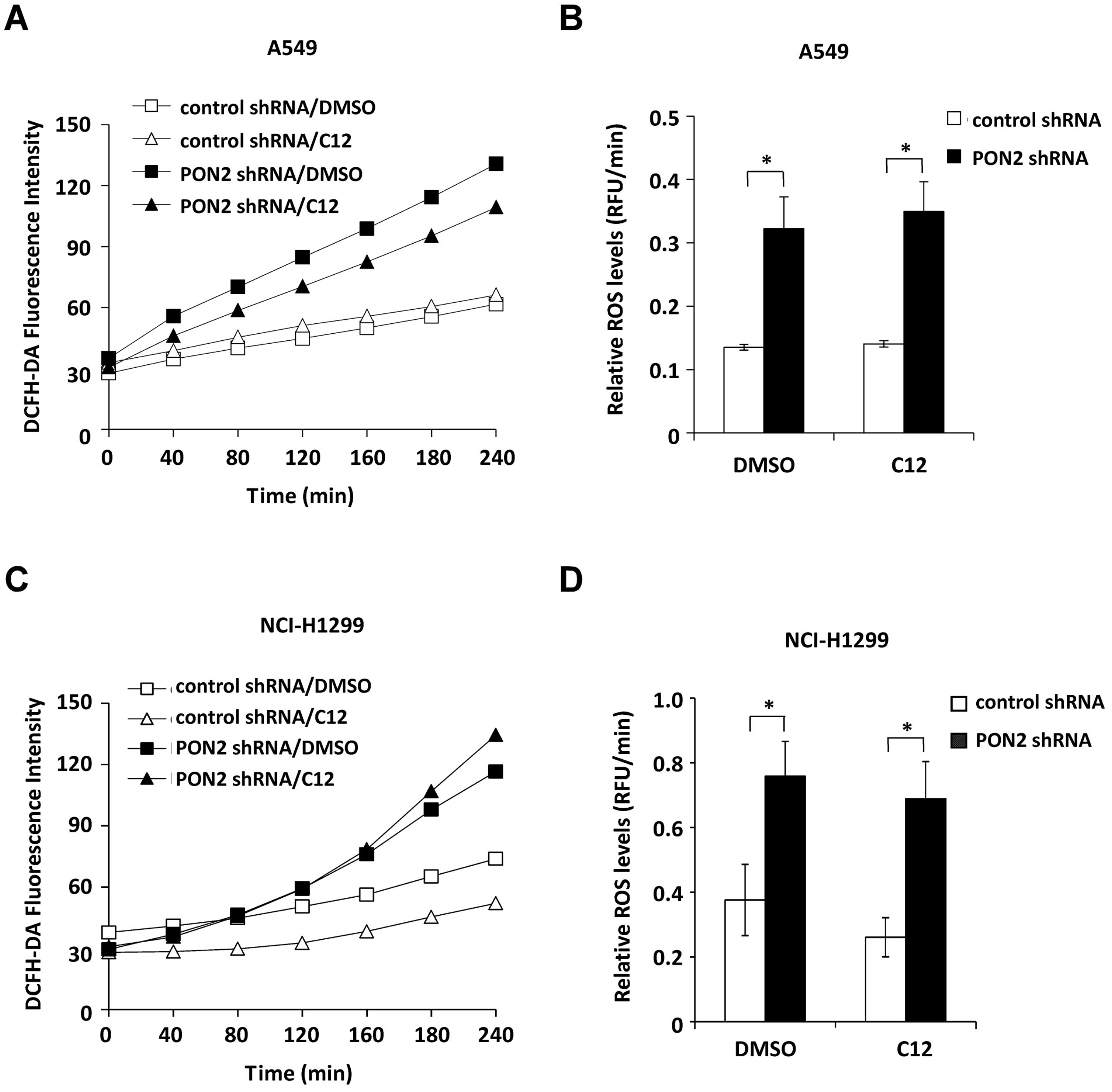


**Supplementary Figure S10: Endogenous PON2 possesses antioxidant activities in NSCLC cells.** (**A**) Intracellular ROS levels in A549 PON2-knockdown or vector control cells were evaluated by measuring relative fluorescence units (RFUs) of DCFH-DA oxidation by ROS for 4 hours in the presence of absence of 100 μM C12. Representative results are shown. (**B**) The data shown in (A) were plotted as RFUs versus time. The relative ROS levels were presented as the values of the slope (RFU/min). The data are mean ± standard deviation of three independent experiments. Asterisks indicate *P* values of < 0.05 (*) by student's unpaired t test. (**C**) The ROS levels of the indicated NCI-H1299 cells were measured following the treatment of 100 μM C12. Typical data are presented. (**D**) The summary of the data shown in (C). The values of the slope (RFU/min) represented the relative ROS levels. Data are shown as mean ± standard deviation of three independent experiments. Asterisks indicate P values of < 0.05 (*) by student's unpaired t test.

## SUPPLEMENTARY VIDEOS

**Supplementary Video S1: Time-lapse recording of mitochondrial matrix-targeted GFP fluorescence in A549 cells treated with the vehicle control DMSO**

**Supplementary Video S2: Time-lapse recording of TMRE fluorescence in A549 cells treated with the vehicle control DMSO**

**Supplementary Video S3: Time-lapse recording of mitochondrial matrix-targeted GFP fluorescence in A549 cells treated with C12**

**Supplementary Video S4: Time-lapse recording of TMRE fluorescence in A549 cells treated with C12**
